# Adaptation to seasonal reproduction and environment‐associated factors drive temporal and spatial differentiation in northwest Atlantic herring despite gene flow

**DOI:** 10.1111/eva.13675

**Published:** 2024-03-14

**Authors:** Angela P. Fuentes‐Pardo, Ryan Stanley, Christina Bourne, Rabindra Singh, Kim Emond, Lisa Pinkham, Jenni L. McDermid, Leif Andersson, Daniel E. Ruzzante

**Affiliations:** ^1^ Department of Biology Dalhousie University Halifax Nova Scotia Canada; ^2^ Department of Medical Biochemistry and Microbiology Uppsala University Uppsala Sweden; ^3^ Fisheries and Oceans Canada Maritimes Region Dartmouth Nova Scotia Canada; ^4^ Fisheries and Oceans Canada Northwest Atlantic Fisheries Centre St John's Newfoundland and Labrador Canada; ^5^ Fisheries and Oceans Canada St. Andrews Biological Station St. Andrews New Brunswick Canada; ^6^ Fisheries and Oceans Canada Maurice Lamontagne Institute Mont‐Joli Quebec Canada; ^7^ Department of Marine Resources West Boothbay Harbor Maine USA; ^8^ Fisheries and Oceans Canada Gulf Fisheries Centre Moncton New Brunswick Canada; ^9^ Department of Veterinary Integrative Biosciences Texas A&M University College Station Texas USA

**Keywords:** chromosomal inversion, fisheries, genomics, marine fish, pool‐seq, whole genome

## Abstract

Understanding how marine organisms adapt to local environments is crucial for predicting how populations will respond to global climate change. The genomic basis, environmental factors and evolutionary processes involved in local adaptation are however not well understood. Here we use Atlantic herring, an abundant, migratory and widely distributed marine fish with substantial genomic resources, as a model organism to evaluate local adaptation. We examined genomic variation and its correlation with environmental variables across a broad environmental gradient, for 15 spawning aggregations in Atlantic Canada and the United States. We then compared our results with available genomic data of northeast Atlantic populations. We confirmed that population structure lies in a fraction of the genome including likely adaptive genetic variants of functional importance. We discovered 10 highly differentiated genomic regions distributed across four chromosomes. Nine regions show strong association with seasonal reproduction. One region, corresponding to a known inversion on chromosome 12, underlies a latitudinal pattern discriminating populations north and south of a biogeographic transition zone on the Scotian Shelf. Genome–environment associations indicate that winter seawater temperature best correlates with the latitudinal pattern of this inversion. The variation at two so‐called ‘islands of divergence’ related to seasonal reproduction appear to be private to the northwest Atlantic. Populations in the northwest and northeast Atlantic share variation at four of these divergent regions, simultaneously displaying significant diversity in haplotype composition at another four regions, which includes an undescribed structural variant approximately 7.7 Mb long on chromosome 8. Our results suggest that the timing and geographic location of spawning and early development may be under diverse selective pressures related to allelic fitness across environments. Our study highlights the role of genomic architecture, ancestral haplotypes and selection in maintaining adaptive divergence in species with large population sizes and presumably high gene flow.

## 1 INTRODUCTION

Understanding how organisms adapt to their habitat and identifying which genes and environmental factors underpin this process are key questions in evolutionary biology and conservation. Such knowledge can help illustrate how biological diversification occurs (Nosil & Feder, 2012) and provides a genetic framework to direct management actions towards the conservation of intra‐specific diversity (Hohenlohe et al., 2021). In marine species with high gene flow and large population sizes, it has been difficult to establish the extent of population structure and local adaptation by only analysing neutral genetic markers (Hauser & Carvalho, 2008) or a small fraction of the genome. However, with the ability to examine both neutral and adaptive genetic variation through high‐throughput sequencing, recent genomic studies have revealed high levels of genetic differentiation at loci putatively under selection in marine species with seemingly undifferentiated populations at neutral loci [e.g. Atlantic herring (Lamichhaney et al., 2012), cod (Johansen et al., 2020), American lobster (Dorant et al., 2022)].

In theory, gene flow can counteract adaptation by introducing maladaptive genetic variants into locally adapted populations, but it can also bring alleles that may prove beneficial in the local environment (Lenormand, 2002). This apparent paradox begs the question as to how barriers to gene flow may arise in the sea. Studies based on empirical evidence or demographic modelling indicate that gene flow in marine species can be restricted by ecological, geographical, physical and intrinsic factors (Palumbi, 1994). Ecological factors include differences in life history and physiological tolerance (e.g. timing of reproduction or thermal preference/avoidance) (Lowerre‐Barbieri et al., 2011) and in adult reproduction and behavioural phenotype favouring natal homing (Thorrold, 2001) or local recruitment (Levin, 2006). Geographic distance between populations and physical barriers, such as differences in oceanographic conditions (e.g. gradients in salinity or temperature or divergent ocean currents), may limit spatial dispersal of individuals and thus influence gene flow. Intrinsic incompatibilities evolving as a by‐product of divergent selection or historical divergence can also impede gene flow (Hendry et al., 2004; Nosil et al., 2005). In marine species with large effective population sizes (*N*_e_), the role of genetic drift is assumed to be minor, facilitating the detection of signatures of selection. Therefore, abundant migratory marine species inhabiting diverse environments offer ideal systems for the study of ecological adaptation with gene flow. Such is the case of Atlantic herring (Lamichhaney et al., 2017).

Atlantic herring is a pelagic fish that inhabits temperate waters of the north Atlantic, including fully marine environments, fjords and the brackish waters of the Baltic Sea. It forms large schools, often comprising millions of individuals. Juveniles and adults undertake extensive annual migrations between feeding, overwintering and spawning areas. In the northwest (NW) Atlantic, spawning occurs from southern Labrador to the Gulf of Maine at relatively predictable times and locations near shore, peaking in spring (April–May) and fall (September–October) (McQuinn, 1997; Stephenson et al., 2009; Wheeler & Winters, 1984). Diversity in reproductive phenology and spawning behaviour can promote temporal and spatial reproductive isolation between populations that are likely exposed to contrasting environments and selective pressures. These characteristics make this species an ideal model for investigating the genetic basis and mechanisms involved in ecological adaptation.

Herring plays a critical role in the marine food chains as a forage species that feeds on plankton and is an important food source for other species of fish, mammals and sea birds. In addition, herring populations sustain large fisheries throughout the North Atlantic (FAO, 2019), some of which have experienced periods of severe decline in the last century (Engelhard & Heino, 2004; Overholtz, 2002; Simmonds, 2007). In the NW Atlantic, declining abundance has impacts on both commercial landings and the various fisheries that use forage fish such as herring and Atlantic mackerel (*Scomber scombrus*) as bait. This is the case of American lobster (*Homarus americanus*), which has become the most valuable fishery resource in North America. In 2022, following a lack of recovery, Fisheries and Oceans Canada (DFO) announced a moratorium on directed commercial and bait fisheries for the spring stock of Atlantic herring in the southern Gulf of St. Lawrence (DFO, 2022). Although the status of individual stocks is variable across the Atlantic Canadian region (e.g. from cautious to critical in the Gulf of St. Lawrence, or stable in Newfoundland), herring abundance overall has been decreasing or sustained at low levels, which is largely attributed to high adult mortality and low recruitment (Turcotte et al., 2021). Therefore, in recent years there has been increased interest in understanding the population dynamics of herring stocks using diverse approaches, including genetics.

Numerous research efforts have aimed to estimate the level of population structure of herring using various genetic markers and at different spatial scales, primarily in the northeast (NE) Atlantic. Initial genetic studies analysed a few dozen of neutral markers and reported panmixia or low population differentiation (Andersson et al., 1981; André et al., 2011; Bekkevold et al., 2005; Jørgensen et al., 2005; Ruzzante et al., 2006). Subsequent studies based on genetic variants derived from the transcriptome (Limborg et al., 2012) or reduced representation sequencing (Guo et al., 2016) and studies that used genome sequencing with an exome assembly (Lamichhaney et al., 2012) showed that genetic differentiation occurs at outlier loci likely associated with environmental gradients in the Baltic Sea. Whole‐genome studies using a scaffolded reference genome revealed a number of genes associated with ecological adaptation (Martinez Barrio et al., 2016). The recent assembly of a chromosome‐level reference genome for the species has opened the possibility to examine structural rearrangements and more complete gene models (Pettersson et al., 2019), a breakthrough in the ability to study the genetic basis of adaptation in this species. The most recent whole‐genome study analysed 53 locations, 48 from the NE and 5 from the NW Atlantic, and disclosed hundreds of loci underlying ecological adaptation (Han et al., 2020). About 30 loci showed consistent association with adaptation to salinity, seven to spawning time and four chromosomal inversions are presumably related to adaptation to seawater temperature during spawning (Han et al., 2020; Jamsandekar et al., 2023). Candidate genes associated with spawning time include the thyroid‐stimulating hormone receptor (*tshr*), *sox11b* transcription factor (*sox11b*), calmodulin (*calm1b*) and oestrogen receptor 2 (*esr2a*), all located on chromosome (Chr) 15 and with a known or presumed role in reproductive biology. The gene *myhc* (myosin heavy chain) on Chr12 is putatively involved in myogenesis and plasticity of seasonal development in herring (Johnston et al., 2001).

Compared to the NE Atlantic, fewer research initiatives have focused on the structure of NW herring populations. Earlier studies analysed nine microsatellite markers and found low but significant genetic differentiation between populations (McPherson et al., 2001, 2003, 2004). One study compared samples collected at the same location 10 years apart and found temporal stability of allele frequencies at a subset of SNPs related with spawning time (Kerr et al., 2019). More recent whole‐genome studies, primarily focused on NE Atlantic populations and including the same five Canadian populations (Han et al., 2020; Lamichhaney et al., 2017), found low genome‐wide differentiation at presumed neutral loci but high divergence at outlier loci. Only about 25% of SNPs associated with seasonal reproduction showed equivalent genetic variation in NW and NE Atlantic populations (Lamichhaney et al., 2017). The fact that a large proportion of outlier loci are likely not shared between populations across the ocean (~75%) brings to question whether herring display local adaptation to NW Atlantic environments, though historical contingencies could also be at least partially responsible for the differences observed in rates of gene flow between seasonal spawning types in the NW and NE Atlantic populations (see Fang et al., 2020).

Here, we test the hypothesis of local adaptation through an exhaustive examination of genome‐wide variation of 15 spawning aggregations of herring in the NW Atlantic. Where appropriate, we compare patterns observed in this study with those described previously for NE Atlantic herring populations. In particular, we address three focal questions: (1) What is the temporal and spatial scale of population structuring in the target area? (2) What is the genomic basis of population divergence and local adaptation? (3) Which evolutionary mechanisms and environmental factors are likely involved in population divergence? We integrate pooled DNA whole‐genome sequencing (pool‐seq) and oceanographic data to address these questions. Given the role of temperature and photoperiod in regulating the onset of seasonal reproduction in numerous temperate fish species (Migaud et al., 2010) and the relatively high predictability of spawning season and location in Atlantic herring (Sinclair & Tremblay, 1984), we examined whether the genetic differences among spawning aggregations are likely more attributable to natural selection or demographic history.

## 2 MATERIALS AND METHODS

### 2.1 Study area and sample collection

**FIGURE 1:**
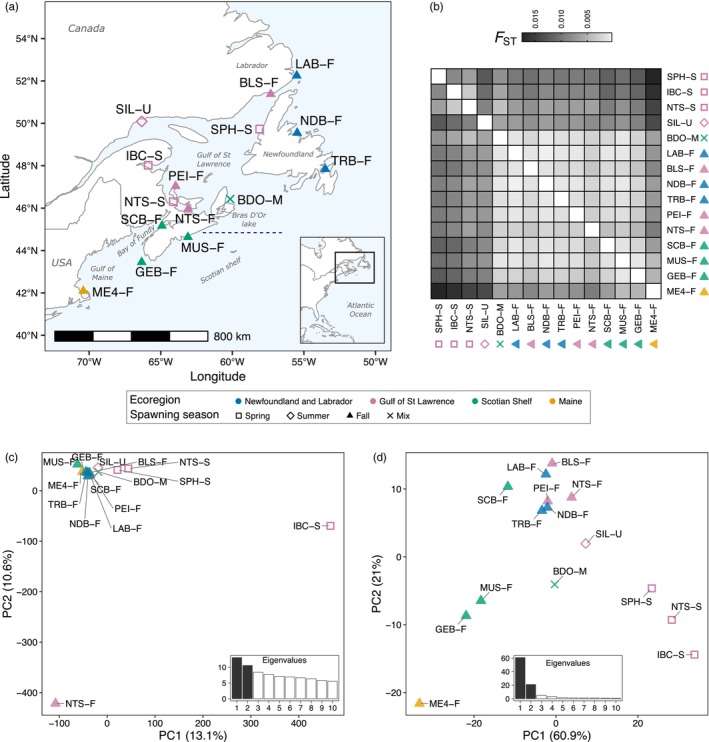
Sampling locations and population structure of northwest Atlantic herring. (a) Map depicting collection sites. Location name abbreviations as described in Table 1, in which the spawning season is indicated with the suffix ‘‐S’ for spring, ‘‐F’ for fall, ‘‐U’ for summer and ‘‐M’ for mixed. Each point corresponds to a pool sample. Point colours indicate the designation of the sample to one of the major biogeographic units described for the Canadian Atlantic Ocean. Symbol shapes represent the predominant spawning season based on individual gonadal maturation status at the time of collection (Figure S1). The horizontal blue dashed line on the map indicates the approximate location of a biogeographic transition zone in the eastern Scotian Shelf, 44.61° N ± 0.25 (Stanley et al., 2018). (b) Heatmap plot representing pairwise *F*_ST_ estimates based on pool allele frequencies of 5,073,572 SNPs (Table S2). Samples are ordered by season, and within season, by latitude. Cell shading represents the degree of genomic divergence between a pair of pool samples, which goes from a lack (white) to high (black) differentiation. Principal component analysis plot based on undifferentiated (c) (*n* = 135,950) and highly differentiated (d) markers (*n* = 545). In both plots the first two axes or principal components (PCs) are shown and the inset bar plots indicate the percentage of the variance explained by each of the first 10 PCs. Same as before, each point corresponds to a pool sample, its colour indicates the designated biogeographic unit and its shape, the corresponding spawning season.

The study area comprises the reproductive range of *Clupea harengus* in the NW Atlantic, from southern Labrador in Canada to the Gulf of Maine in the United States (Figure 1 and Table 1). We collected ~50 individuals from 10 locations across this area. Sample collection took place between 2012 and 2016 during or near the peak of spawning at each site, mainly in spring (April–May), summer (June) and fall (August–October) seasons. Individuals actively spawning or ready to spawn (gonadal maturity stages 5 and 6 respectively) were targeted, when possible, to have a representative sample of distinct reproductive units (or populations) and to minimize the chance of sampling non‐spawning migrants. Most of the fish were actively spawning or ready to spawn, except in the Bras D'Or lake (BDO‐M), in which only 15% of fish were mature and the rest were in resting condition (Figure S1). Therefore, this sample was considered mixed. Gonadal maturity was visually assessed following classifications of Bucholtz et al. (2008). Muscle or fin tissues were collected from each individual and preserved in 95% ethanol at −20°C until processing.

**TABLE 1:** Herring samples included in this study, corresponding to 15 pools and 45 individuals collected in the NW Atlantic.

Locality	ID code	Sample size (*N*)	Latitude	Longitude	Sampling date	Spawning season	Source	BioSample accession
Pool data								
Seven Islands (Sept‐Îles)	SIL‐U	50	50.09	−66.33	06 June 2012	Summer	This study	SAMN33007718
Inner Bay Chaleur	IBC‐S	41	48.00	−65.85	08 May 2014	Spring	Lamichhaney et al. (2017), WInB	SAMN05589078
Stephenville	SPH‐S	48	49.73	−57.94	30 May 2012	Spring	This study	SAMN33007720
Northumberland Strait	NTS‐S	49	46.30	−64.12	06 May 2014	Spring	Lamichhaney et al. (2017), WNsS	SAMN05589080
Northumberland Strait	NTS‐F	50	45.73	−62.60	16 September 2014	Fall	Lamichhaney et al. (2017), WNsF	SAMN05589079
Labrador	LAB‐F	50	52.25	−55.50	24 August 2014, 22 August 2015	Fall	This study	SAMN33007723
Blanc Sablon	BLS‐F	49	51.38	−57.31	13 August 2014	Fall	This study	SAMN33007724
Notre Dame Bay	NDB‐F	50	49.55	−55.47	26 October 2015	Fall	This study	SAMN33007725
Trinity Bay	TRB‐F	49	47.84	−53.47	28 September 2014	Fall	Lamichhaney et al. (2017), WBob	SAMN05589075
Prince Edward Island	PEI‐F	50	47.04	−63.96	25 August 2014	Fall	This study	SAMN33007727
Bras D'Or lake	BDO‐M	50	45.93	−60.85	20 April 2016	Spring	This study	SAMN33007728
Scots Bays	SCB‐F	50	45.17	−64.92	24 August 2015	Fall	This study	SAMN33007729
Musquodoboit	MUS‐F	50	44.63	−63.10	28 October 2015	Fall	This study	SAMN33007730
German Banks	GEB‐F	48	43.16	−66.18	28 August 2014	Fall	Lamichhaney et al. (2017), WGeB	SAMN05589077
Maine fishing area 514	ME4‐F	50	42.09	−70.41	19 October 2015	Fall	This study	SAMN33007732
Individual data								
Blanc Sablon	14F4Sbs344_Canada_Atlantic_Autumn	14F4Sbs344	51.38	−57.31	2014	Autumn	This study	SAMN39750335
Blanc Sablon	14F4Sbs349_Canada_Atlantic_Autumn	14F4Sbs349	51.38	−57.31	2014	Autumn	This study	SAMN39750336
Prince Edward Island	14F4TL404_Canada_Atlantic_Autumn	14F4TL404	47.04	−63.96	2014	Autumn	This study	SAMN39750337
Prince Edward Island	14F4TL415_Canada_Atlantic_Autumn	14F4TL415	47.04	−63.96	2014	Autumn	This study	SAMN39750338
Musquodoboit	14F4WK306_Canada_Atlantic_Autumn	14F4WK306	44.63	−63.10	2014	Autumn	This study	SAMN39750339
Musquodoboit	14F4WK316_Canada_Atlantic_Autumn	14F4WK316	44.63	−63.10	2014	Autumn	This study	SAMN39750340
Labrador	15F2J602_Canada_Atlantic_Autumn	15F2J602	52.25	−55.50	2015	Autumn	This study	SAMN39750341
Labrador	15F2J603_Canada_Atlantic_Autumn	15F2J603	52.25	−55.50	2015	Autumn	This study	SAMN39750342
Labrador	15F2J606_Canada_Atlantic_Autumn	15F2J606	52.25	−55.50	2015	Autumn	This study	SAMN39750343
Labrador	15F2J616_Canada_Atlantic_Autumn	15F2J616	52.25	−55.50	2015	Autumn	This study	SAMN39750344
Notre Dame Bay	15F3K601_Canada_Atlantic_Autumn	15F3K601	49.55	−55.47	2015	Autumn	This study	SAMN39750345
Notre Dame Bay	15F3K602_Canada_Atlantic_Autumn	15F3K602	49.55	−55.47	2015	Autumn	This study	SAMN39750346
Notre Dame Bay	15F3K609_Canada_Atlantic_Autumn	15F3K609	49.55	−55.47	2015	Autumn	This study	SAMN39750347
Notre Dame Bay	15F3K621_Canada_Atlantic_Autumn	15F3K621	49.55	−55.47	2015	Autumn	This study	SAMN39750348
Scots Bays	15F4XRsb625_Canada_Atlantic_Autumn	15F4XRsb625	45.17	−64.92	2015	Autumn	This study	SAMN39750349
Scots Bays	15F4XRsb626_Canada_Atlantic_Autumn	15F4XRsb626	45.17	−64.92	2015	Autumn	This study	SAMN39750350
Gulf of Maine	15F5Y514–617_US_Atlantic_Autumn	15F5Y514‐617	42.09	−70.41	2015	Autumn	This study	SAMN39750351
Gulf of Maine	15F5Y514–620_US_Atlantic_Autumn	15F5Y514‐620	42.09	−70.41	2015	Autumn	This study	SAMN39750352
Bonavista Bay	F3L312_Canada_Atlantic_Autumn	F3L312	48.82	−53.33	2014	Autumn	Lamichhaney et al. (2017)	SAMN05589090
Bonavista Bay	F3L337_Canada_Atlantic_Autumn	F3L337	48.82	−53.33	2014	Autumn	Lamichhaney et al. (2017)	SAMN05589091
Northumberland Strait	F4TH327_Canada_Atlantic_Autumn	F4TH327	45.73	−62.60	2014	Autumn	Lamichhaney et al. (2017)	SAMN05589098
Northumberland Strait	F4TH337_Canada_Atlantic_Autumn	F4TH337	45.74	−62.60	2014	Autumn	Lamichhaney et al. (2017)	SAMN05589099
German Banks	F4XQgb402_Canada_Atlantic_Autumn	F4XQgb402	43.27	−66.30	2014	Autumn	Lamichhaney et al. (2017)	SAMN05589094
German Banks	F4XQgb408_Canada_Atlantic_Autumn	F4XQgb408	43.27	−66.30	2014	Autumn	Lamichhaney et al. (2017)	SAMN05589095
Stephenville	12S4Rsv38_Canada_Atlantic_Spring	12S4Rsv38	49.73	−57.94	2012	Spring	This study	SAMN39750353
Stephenville	12S4Rsv41_Canada_Atlantic_Spring	12S4Rsv41	49.73	−57.94	2012	Spring	This study	SAMN39750354
Fortune Bay	14S3Ps233_Canada_Atlantic_Spring	14S3Ps233	47.52	−55.40	2014	Spring	This study	SAMN39750355
Fortune Bay	14S3Ps265_Canada_Atlantic_Spring	14S3Ps265	47.52	−55.40	2014	Spring	This study	SAMN39750356
Notre Dame Bay	15S3K404_Canada_Atlantic_Spring	15S3K404	49.55	−55.44	2015	Spring	This study	SAMN39750357
Notre Dame Bay	15S3K410_Canada_Atlantic_Spring	15S3K410	49.55	−55.44	2015	Spring	This study	SAMN39750358
Notre Dame Bay	15S3K430_Canada_Atlantic_Spring	15S3K430	49.55	−55.44	2015	Spring	This study	SAMN39750359
Notre Dame Bay	15S3K449_Canada_Atlantic_Spring	15S3K449	49.55	−55.44	2015	Spring	This study	SAMN39750360
Placentia Bay	16S4Pla78_Canada_Atlantic_Spring	16S4Pla78	47.31	−53.94	2016	Spring	This study	SAMN39750361
Placentia Bay	16S4Pla79_Canada_Atlantic_Spring	16S4Pla79	47.31	−53.94	2016	Spring	This study	SAMN39750362
Placentia Bay	16S6Pla71_Canada_Atlantic_Spring	16S6Pla71	47.31	−53.94	2016	Spring	This study	SAMN39750363
Placentia Bay	16S6Pla72_Canada_Atlantic_Spring	16S6Pla72	47.31	−53.94	2016	Spring	This study	SAMN39750364
Bras D'Or lake	16SBDO106_Canada_Atlantic_Spring	16SBDO106	45.93	−60.85	2016	Spring	This study	SAMN39750365
Bras D'Or lake	16SBDO107_Canada_Atlantic_Spring	16SBDO107	45.93	−60.85	2016	Spring	This study	SAMN39750366
Fortune Bay	S3Ps246_Canada_Atlantic_Spring	S3Ps246	47.28	−55.63	2014	Spring	Lamichhaney et al. (2017)	SAMN05589092
Northumberland Strait	S4TH231_Canada_Atlantic_Spring	S4TH231	46.32	−64.15	2014	Spring	Lamichhaney et al. (2017)	SAMN05589100
Northumberland Strait	S4TH244_Canada_Atlantic_Spring	S4TH244	46.32	−64.15	2014	Spring	Lamichhaney et al. (2017)	SAMN05589101
Inner Baie Des Chaleurs	S4TM205_Canada_Atlantic_Spring	S4TM205	48.00	−65.85	2014	Spring	Lamichhaney et al. (2017)	SAMN05589096
Inner Baie Des Chaleurs	S4TM211_Canada_Atlantic_Spring	S4TM211	48.00	−65.85	2014	Spring	Lamichhaney et al. (2017)	SAMN05589097
Seven Islands	12S4S7i22_Canada_Atlantic_Summer	12S4S7i22	50.09	−66.33	2012	Summer	This study	SAMN39750367
Seven Islands	12S4S7i28_Canada_Atlantic_Summer	12S4S7i28	50.09	−66.33	2012	Summer	This study	SAMN39750368

### 2.2 DNA extraction, pool and individual sequencing

Total genomic DNA was isolated from individual tissues using a standard phenol–chloroform protocol (Sambrook & Russell, 2006). DNA concentration (in nanograms per microlitre, ng/μL) was measured in triplicates using the Quant‐iT PicoGreen dsDNA assay (Thermo Fisher Scientific) and the Roche LightCycler 480 Instrument (Roche Molecular Systems, Inc., Germany). DNA integrity was inspected using 0.8% agarose gel electrophoresis with 0.5× TBE buffer and a 1 kilo base pairs (kbp) molecular weight ladder.

To assess genome‐wide variation of each spawning aggregation as a whole, we used the pool DNA whole‐genome sequencing (pool‐seq) approach. This technique involves sequencing the combined DNA of several individuals from a population using a single barcoded library to generate population‐level whole‐genome data for a fraction of the cost of sequencing individuals to high depth, at the expense of missing individual genotypes (Schlötterer et al., 2014). We aimed to mix in a pool equal amount of DNA of ~40 to 50 individuals collected at a given spawning site and season. Individual DNA were normalized to a common concentration of 30 ng/μl and combined to a single tube using the liquid handling robot epMotion 5407 (Eppendorf, Germany). To characterize haplotype patterns of loci of interest, we generated high coverage whole‐genome sequence data for 1–4 individuals per location for a total of 34 individuals distributed across 14 locations (Table 1). Sequencing library preparation and high‐throughput sequencing of both pool and individual DNA samples were performed by The Centre for Applied Genomics of the Hospital for Sick Children, Canada. A TruSeq Nano Illumina DNA library with an insert size of 550 bp was built for each DNA pool and individual. Illumina paired end reads (2 × 126 bp) were generated for each library with a HiSeq‐2500 sequencer. The target average depth of coverage per pool was 50× and per individual was 20×. We complemented our pool and individual datasets with publicly available raw sequence data of 5 Canadian pools and of 11 individuals (Lamichhaney et al., 2017) (Table 1). Therefore, the NW Atlantic dataset was represented by a total of 15 pools and 45 individuals.

### 2.3 Sequence filtering, alignment and variant calling

Sequence quality of raw reads per pool and individual was examined with *FastQC* v0.11.5 (Andrews, 2010), and a single report for all pools or individuals was generated with *MultiQC* (Ewels et al., 2016). Illumina adapters, low‐quality bases (Phred score < 20), reads shorter than 40 bp and single‐end reads were removed from the datasets using *Trimmomatic* v.0.36 (Bolger et al., 2014) [parameters: ILLUMINACLIP:TruSeq3‐PE‐2.fa:2:30:10 SLIDINGWINDOW:5:20 MINLEN:40].

The remaining high‐quality paired end sequences were mapped against the chromosome‐level genome assembly of Atlantic herring (Pettersson et al., 2019) using the Burrows‐Wheeler Aligner (*BWA*) v0.7.12‐r1039 [default parameters, MEM algorithm] (Li, 2013). Read mapping quality was assessed with *Qualimap* v.2.2.1 (Okonechnikov et al., 2015). Mapped reads in the form of BAM files were sorted, PCR duplicates marked and read groups added using *Picard tools* v2.10.2 (Broad Institute, 2018). An index file for each BAM file was generated with *SAMtools* v1.5 (Li & Durbin, 2009).

To call genetic variants in the pools we used *UnifiedGenotyper* and, in the individuals, *HaplotypeCaller*, both algorithms implemented in the program *GATK* v3.8 (McKenna et al., 2010). Indels were removed and biallelic SNPs were kept for further filtering. We applied various filters to retain high confidence variants. We required that SNPs passed *GATK* variant quality filters, which assess sequence and mapping quality, strand bias and variant position on reads. For the pool data, we established cut‐off values for the *GATK* quality filters based on density plots (Figure S2) [filters applied: FS > 60.0, SOR > 3.0, MQ < 40.0, MQRankSum < −12.5, ReadPosRankSum < −8.0; (for a description of each annotation see Broad Institute, 2016)]. Using *BCFtools* (Danecek et al., 2021), we retained SNPs that had a missing rate <20%, a minimum minor allele count of 2 and were polymorphic. We retained the SNPs within the 5–99 percentile of the coverage distribution per pool (Figure S3), thus excluding spurious SNPs in repetitive regions and copy number variants that commonly have excessively high coverage. For the individual data, we required that SNPs passed GATK hard filtering, had an average depth of coverage (DP) between 8 and 160×, genotype quality (GQ) >30, were polymorphic, had a minor allele frequency (MAF) >0.1 and missing rate <10%.

Allele frequencies in pool‐seq data are derived from the read counts of each allele per SNP. Read counts can vary among pools and along the genome due to technical biases during sequencing (Dohm et al., 2008; Kolaczkowski et al., 2011). To account for sampling error of the pool during sequencing before calculating allele frequencies, we rescaled the raw read counts to the effective sample size *n*_eff_ using a python script that implemented the formula:

$$n_{\mathrm{eff}}=\frac{\left(n\times \mathrm{RD}-1\right)}{n+\mathrm{RD}}$$

where RD is the raw read depth and *n* is the number of chromosomes in a pool (e.g. *n* = 2 × *number of individuals in a pool*, for diploid species). *n*_eff_ represents an estimate of the effective number of chromosomes in a pool adjusted by the read depth (Bergland et al., 2014; Feder et al., 2012; Kolaczkowski et al., 2011). Based on the rescaled read counts, we calculated allele frequencies per SNP for each pool.

### 2.4 Population structure and genetic diversity

We estimated the genomic differentiation between pools using pairwise *F*_ST_ and principal components analysis (PCA). We computed the unbiased *F*_ST_ for pools (${\hat{F}}_{\mathrm{ST}}^{\text{pool}}$) between all pairs of spawning aggregations using the *R* package *poolfstat* (Hivert, 2018). This algorithm calculates *F*‐statistics equivalent to Weir and Cockerham (1984) and accounts for sampling error in pool‐seq.

To compare population structure patterns of undifferentiated (presumed neutral) and highly differentiated loci (presumed under selection), we separately performed a PCA on two SNP sets defined from the empirical distribution and standard deviation (SD) of allele frequencies of all markers, as applied in Han et al. (2020). The set of undifferentiated markers corresponded to SNPs with allele frequency SD below and close to the mean value (0.03 < allele frequency SD ≤ 0.09), while the highly differentiated markers had an allele frequency SD ≥ 0.2 from the mean (Figure S4). To reduce the effect of physical linkage among loci and considering that in Atlantic herring the average decay of linkage disequilibrium between loci is ~100 bp (*r* ^2^ < 0.1) (Martinez Barrio et al., 2016), we retained one SNP every 1 kbp for the undifferentiated markers and one SNP every 10 kbp for the differentiated markers assuming higher linkage in regions under selection.

To characterize the genetic diversity within populations, we estimated the unbiased nucleotide diversity (*π*) and Tajima's *D* for each pool using the program *PoPoolation* 1.2.2 (Kofler et al., 2011). In brief, a pileup file was generated from the BAM file of each pool using *samtools* v.1.10 (Li, 2011). Indels and SNPs 5 bp around indels were removed. To account for coverage variation due to sequencing, we sub‐sampled without replacement the depth of coverage of each pileup file to a uniform value, corresponding to the 5% quantile of the coverage distribution (the minimum coverage of a SNP to be retained). SNPs with read depth between 5% and 99% of the empirical coverage distribution per pool, minimum base and mapping quality of 20 and a minor allele count of 2 were retained for further analyses. The statistics *π* and Tajima's *D* were calculated in sliding windows of 10 kbp with a step size of 2 kbp. Windows with a minimum sequencing coverage of 50% were used for further analyses. All statistical tests and graphics were performed in *R* (R Core Development Team, 2023).

### 2.5 Detection of genomic regions likely under selection

To identify outlier genomic regions that are probable targets of selection, we calculated the absolute difference in allele frequencies per SNP (delta allele frequency, dAF) between paired contrasts of pools, using the formula: dAF = absolute[meanAF(group1) − meanAF(group2)]. Based on PCA clustering patterns and prior ecological knowledge of spawning stocks, the two most informative contrasts were established to compare: (i) spring and fall spawners and (ii) northern and southern fall spawners. This analysis was complemented with the calculation of the moving average (or rolling mean) of dAF values over 100 consecutive SNP makers, which smooths out the signal of divergence while ruling out the effect of single outlier SNPs that might result from sequencing artefacts.

To identify loci that are likely related with local adaptation in the NW Atlantic, we compared the outlier SNPs found in this study with those reported in Han et al. (2020), showing consistent association with spawning time and salinity adaptation and within four putative inversions (on Chr6, 12, 17 and 23). To examine the genetic patterns of genomic regions of interest in Atlantic herring populations and its sister species, Pacific herring (*Clupea pallasii*), we combined our data of 15 NW Atlantic pools with publicly available data of 47 pools from the Baltic Sea and the NE Atlantic and one pool of Pacific herring from Vancouver, Canada (Han et al., 2020), for a total of 63 pools (Table S1). We visually inspected the allele frequencies of outlier SNPs per region using a heatmap plot. Additionally, we generated a neighbour‐joining (NJ) tree based on the allele frequencies of outlier SNPs per region using the R package *ape*. The distance matrix used for the NJ tree corresponded to the outer product of the arrays derived from the sum of the absolute difference in allele frequency (dAF) for all positions normalized by the number of positions without missing values.

### 2.6 Genome–environment associations

To identify which environmental variables are strongly correlated with outlier SNPs of interest, we performed a redundancy analysis (RDA). The environmental dataset consisted of hours of day light (dayLightHours), sea surface temperature (SST) and salinity (SSS) during the months of spawning at each location and for the winter and summer seasons, representing the coldest and warmest periods of the year respectively. These variables were chosen because they are relevant in fish physiology and have been linked to population structuring of numerous marine species in the NW Atlantic (Stanley et al., 2018). Temperature and salinity values for each sample location were derived from monthly environmental data layers of SST and SSS developed for the North Atlantic between 2008 and 2017 based on the Bedford Institute of Oceanography North Atlantic Model (BNAM), a high‐resolution numerical ocean model. A detailed description of oceanic (Madec et al., 1998) and sea ice (Fichefet & Maqueda, 1997) components of the model can be found in Wang et al. (2016) and Brickman et al. (2018). Data layers were converted to an ASCII grid with a NAD83 projection (ellipse GRS80) and had a nominal resolution of 1/12° (~5 km^2^). Binned data for the months of spawning at each location (spring: April–May, fall: September–October), and for the winter (January–February–March) and summer (July–August–September) seasons, were averaged across 9 years in order to capture long‐term trends of oceanographic variation. Extractions (mean, min and max values) were taken from these averaged layers for each sample location. Prior to performing RDA, environmental data were standardized to zero mean and unit variance using R. Collinearity between environmental variables was assessed using two methods, with pairwise correlation coefficients computed with the function *pairs.panels* of the R package *psych* (Revelle, 2018) (Figure S5) and with variance inflation factors (VIF) obtained from RDA models built with the R package *vegan* (Dixon, 2003). The most collinear variables were removed (|*R* ^2^ ≥ 0.7|) and remaining collinear variables were identified and excluded one by one in consecutive RDA runs based on their VIF value. The variable with the highest VIF was discarded in each run until all variables had a VIF <5, following recommendations of Zuur et al. (2010). The uncorrelated set of environmental variables (|*R* ^2^ < 0.7|) consisted of summer sea surface temperature (SST_Summer_), winter sea surface temperature (SST_Winter_), sea surface temperature during spawning (SST_Spawn_) and day light hours (Figure S6).

For RDA, we used the uncorrelated set of environmental data as constraining factors for the population allele frequencies of outlier SNPs in genomic regions of interest. RDA runs were performed with the R package *vegan*. Environmental variables that best explained the genetic variance were confirmed with a bi‐directional stepwise permutational ordination method (1000 iterations) using the function *ordistep*. Significance of the overall RDA model and the most explanatory environmental variables was assessed with an analysis of variance (ANOVA) using 9999 permutations.

### 2.7 Linkage disequilibrium decay and allele exchange in SVs

We used the SNP genotypes of Atlantic herring individuals sequenced to high depth to estimate linkage disequilibrium (LD) and the level of allele exchange between haplotypes (gene flux) of the five SVs reported for the species. For the SV in Chr8, we used the 45 NW Atlantic herring individuals. For the inversions in Chr6, 12, 17 and 23, we used an available dataset that includes 79 individuals from the NW and NE Atlantic (Pettersson et al., 2023), as three of these inversions (Chr6, 17 and 23) are monomorphic in the NW Atlantic (Han et al., 2020). To reduce computation time, we performed these analyses on a subset VCF file obtained using *BEDTools* v.2.29.2 (Quinlan & Hall, 2010), containing SNPs within the approximate coordinates of the target region and the flanking region up to ±1 Mbp. With *vcftools* v.0.1.16 (Danecek et al., 2011), we calculated LD as the squared correlation coefficient (*R* ^2^) between genotypes using a 5‐kb window. For comparison, we made this calculation for all individuals and separately for only the homozygote individuals for each of the alternative SV alleles (AA or BB in this case). We visualized the *R* ^2^ values across the SV and in flanking regions using a heatmap plot.

To estimate the extent of allele exchange between the two SV haplotypes, we followed the same procedure applied on the Chr12 putative inversion in Pettersson et al. (2019). In brief, we selected the AA and BB homozygous individuals for the SV. In 10‐kb windows, we classified SNPs as ‘shared’ or ‘diagnostic’ based on their allele frequencies in each group. Markers were categorized as ‘shared’, when they had allele frequencies within the 10%–90% range in both haplotype groups, which is not expected in a canonical inversion with a single origin and completely suppressed recombination. Markers were classified as ‘diagnostic’, when they had an allele frequency >90% in one group and <10% in the other group. We plotted the count of shared and diagnostic SNPs per window using the R package *ggplot2*.

## 3 RESULTS

### 3.1 Whole‐genome sequence data of pools and individuals

We collected individuals from 10 spawning aggregations throughout the NW Atlantic (Figure 1a). We generated pool‐seq data by pooling the DNA of 41–50 individuals per location and generated paired end short reads for the whole genome using a HiSeq Illumina sequencer. Our sequencing effort yielded a total of ~200 Giga base pairs (Gb) of data. The median depth of coverage per pool ranged between 57× and 77× (Table S2). We combined the pool‐seq data of these 10 locations with data of five Canadian locations previously published (Lamichhaney et al., 2017), for a total of 15 pools constituting the dataset of NW Atlantic herring (*n* = 697 individuals). After mapping reads against the genome assembly of the Atlantic herring (Pettersson et al., 2019), calling variants and applying quality filters to the raw variants, we obtained a total of 5,264,683 high‐quality SNPs.

We also generated high‐coverage whole‐genome sequence data of 34 individuals following equivalent sequencing procedures as for the pools, resulting in ~700 Gb of data. The median depth of coverage per individual ranged between 31× and 72× (Table S2). These data were analysed jointly with publicly available data of 11 Canadian samples (Lamichhaney et al., 2017), for a total dataset of 45 individuals. A total of 21,083,037 SNPs passed quality filters and were used in subsequent analyses.

### 3.2 Population genetic structure

We investigated the population structure among samples using pairwise *F*_ST_ and PCA. The *F*_ST_ estimates between pools (${\hat{F}}_{\mathrm{ST}}^{\text{pool}}$) ranged between 0.001 and 0.018 (Figure 1b and Table S3), indicating low genomic differentiation among spawning aggregations across ~1600 km of coastline. This result is consistent with previous studies of Atlantic herring (Han et al., 2020). Despite low differentiation, we detected two subtle patterns of genetic structure between: (i) spring and fall spawners (spring spawners from Stephenville, SPH‐S; Inner Baie Des Chaleurs, IBC‐S; Northumberland Strait, NTS‐S vs. others, mean ${\hat{F}}_{\mathrm{ST}}^{\text{pool}}$ 0.010 ± 0.001); (ii) the southernmost sample in the Gulf of Maine (ME4‐F) and the other fall spawners (mean ${\hat{F}}_{\mathrm{ST}}^{\text{pool}}$ 0.007 ± 0.001).

To evaluate whether population structure patterns were explained mainly by undifferentiated (putatively neutral) or highly differentiated (putatively selective) loci, we separately performed PCA based on the allele frequencies of a subset of neutral (*n* = 135,950, Figure 1c) or outlier markers (*n* = 545, Figure 1d) (see Section 2 for details). In the PCA with neutral markers, most populations formed a single tight cluster with the exception of two pools from the Gulf of St. Lawrence (IBC‐S and NTS‐F), which appeared as outliers. The first two principal components (PCs) explained 23.7% of the genetic variance and this is largely driven by these two pools.

In stark contrast, in the PCA based on presumably adaptive markers, spring and fall spawning populations separated on PC1, and fall spawners separated following a latitudinal pattern along PC2. These two PCs explained a large proportion of the genetic variance (82.0%). On PC2, the ‘northern’ samples included populations from Labrador (LAB‐F), Newfoundland (BLS‐F, TRB‐F, NDB‐F), the Gulf of St. Lawrence (NTS‐F, PEI‐F) and the Bay of Fundy (SCB‐F), while the ‘southern’ samples comprised populations from the Scotian Shelf (GEB‐F, MUS‐F) and the Gulf of Maine (ME4‐F). On PC1, populations from Bras D'Or lake (BDO‐M) and Sept‐Îles (SIL‐U), the first being a mixed sample and the latter collected in the summer, appear to be closer to fall spawning samples. On PC2, spring pools were separated from each other and from the summer pool from Sept‐Îles (SIL‐U), in concordance with the pairwise ${\hat{F}}_{\mathrm{ST}}^{\text{pool}}$ estimates.

### 3.3 Genomic regions likely under selection

Despite low genome‐wide differentiation, we uncovered specific genomic regions displaying high differentiation between some populations. These regions were detected through genome scans based on the absolute difference in allele frequencies (dAF) of 5,264,683 SNPs between pool samples representing distinctive ecotypes or clusters in the PCA. The informative comparisons were: (i) spring versus fall spawners and (ii) northern versus southern fall spawners.

**FIGURE 2:**
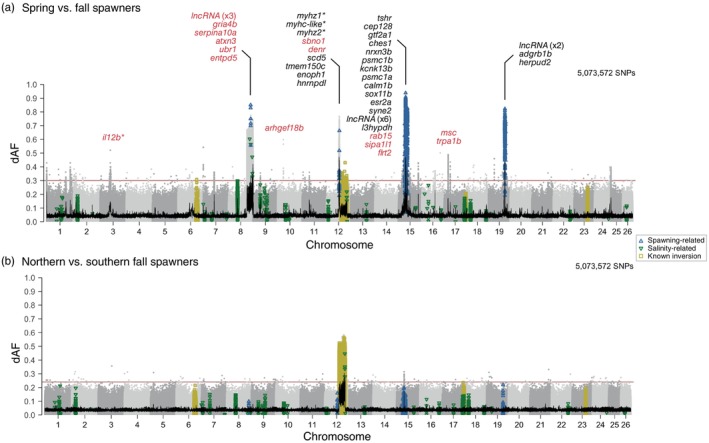
Genomic regions associated with adaptation to seasonal reproduction and a latitudinal environmental cline. Genetic differentiation (dAF) across the genome (a) between spring and fall spawners and (b) between fall spawners in the north versus south of the transition zone in eastern Scotian Shelf (Stanley et al., 2018). Each dot represents a single SNP. SNPs previously reported in Han et al. (2020) as strongly associated with spawning season, salinity and four known inversions (on Chr6, 12, 17 and 23) are denoted as empty blue upward triangles, green downward triangles and yellow squares respectively. Consecutive chromosomes are coloured in intercalating shades of grey. The 100 SNP‐rolling average of dAF is shown as a black line and the Bonferroni significance value across the genome is shown as a horizontal red line. Genes within ±40 kbp of the most divergent SNPs are shown on top of each informative locus. *Gene names with an were inferred from homology with orthologous genes. Gene names coloured in red correspond to genes within the associated genomic region.

**FIGURE 3:**
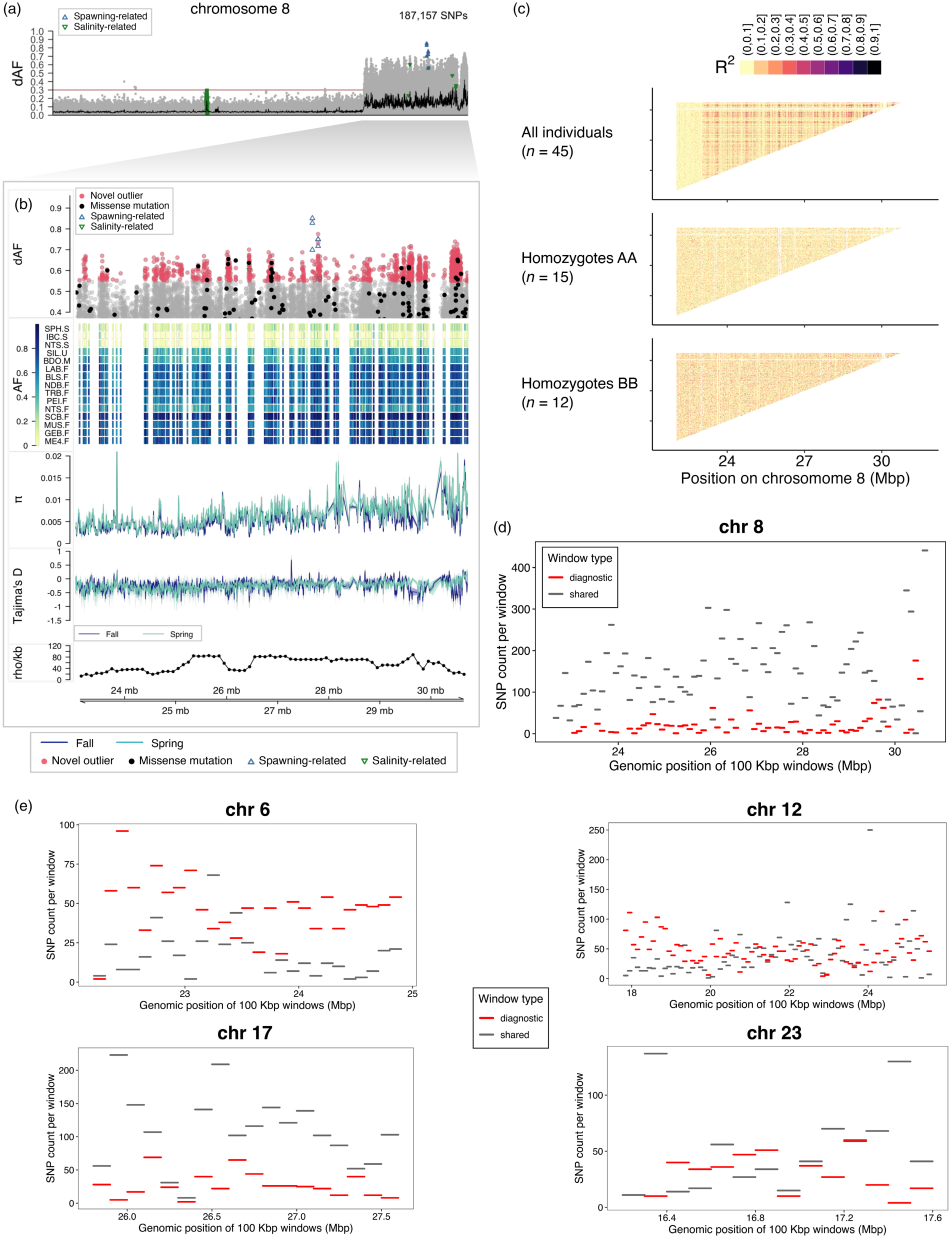
Novel putative inversion on chromosome 8 associated with seasonal reproduction. (a) Genetic differentiation (dAF) between spring and fall spawners across chromosome 8. Each dot is a single SNP. The black line is the rolling average of dAF over 100 SNPs and the horizontal red line is the Bonferroni significance value. SNPs with known association with spawning or salinity (Han et al., 2020) are denoted with empty blue upward triangles or green downward triangles respectively. (b) Close‐up plot to the structural variant (Chr8: 23,040,136–30,729,461). This plot has five tracks, which show from top to bottom: genetic differentiation between spring and fall spawners for SNPs with dAF ≥ 0.4; heatmap plot depicting the minor allele frequency per population (rows) for the novel outlier SNPs (columns); average nucleotide diversity (*π*) and Tajima's *D* (window size 10 kbp, step size 2 kbp) for spring and fall spawners, in light and dark blue lines respectively; and estimate of recombination rate (rho/kbp) every 100 kbp (Pettersson et al., 2019). Novel outlier SNPs (dAF ≥ 0.55) are denoted as red filled circles, missense mutations as filled black circles and other SNPs are grey circles. (c) Linkage disequilibrium pattern among all individuals and among the two types of homozygotes at this putative inversion. Allele sharing for inversion on (d) Chr8, (e) Chr6, 12, 17 and 23.

**FIGURE 4:**
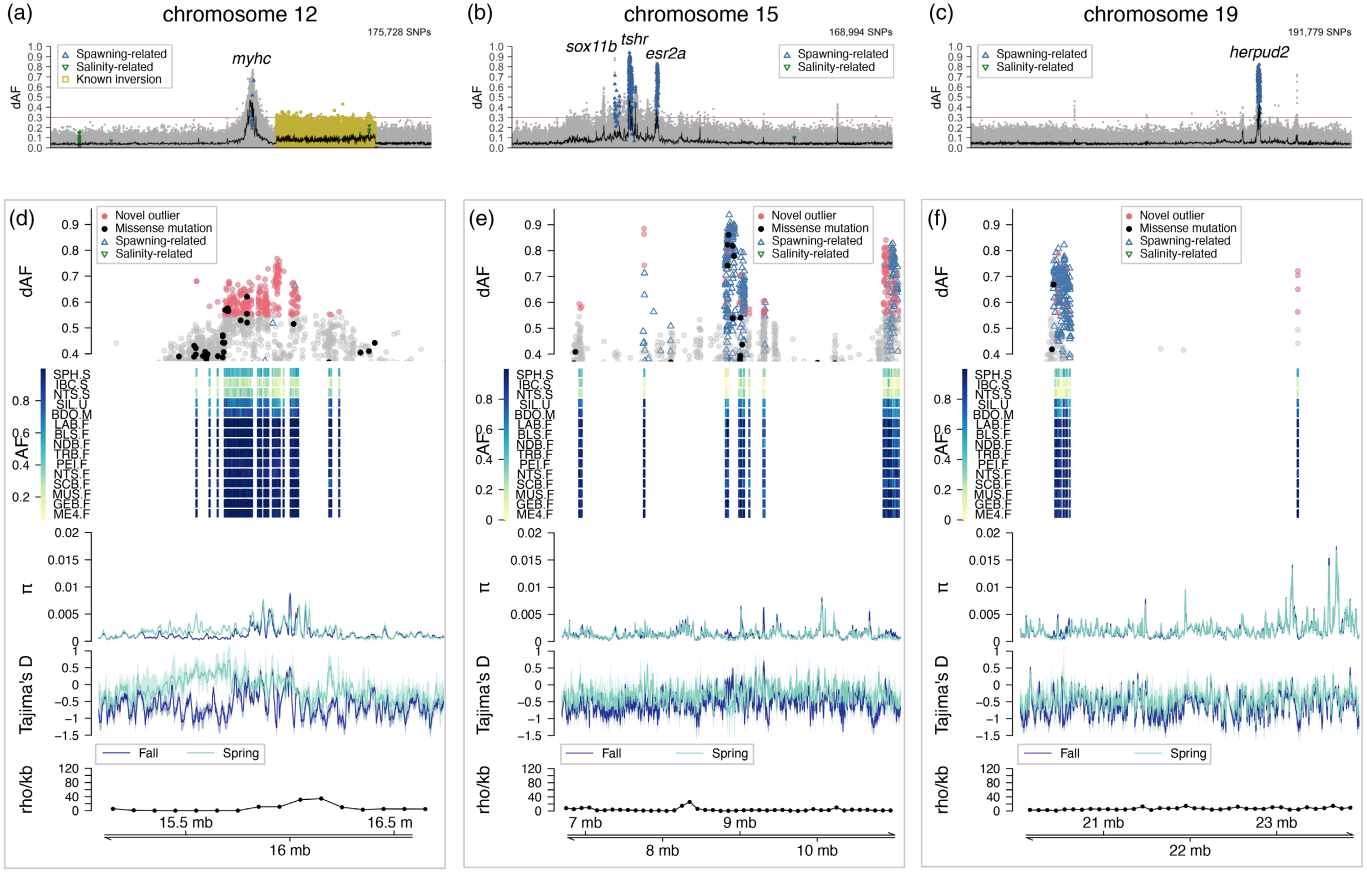
Signatures of selection associated with seasonal reproduction. Genetic differentiation (dAF) between spring and fall spawners across chromosomes (a) 12, (b) 15 and (c) 19. Each dot represents a single SNP. The rolling average of dAF over 100 SNPs is shown as a horizontal black line and the Bonferroni significance value is shown as a horizontal red line. SNPs with described association with spawning, salinity or four inversions (in Chr6, 12, 17 and 23) in Han et al. (2020) are indicated with empty blue upward triangles, green downward triangles and yellow squares respectively. Close‐up to the signatures of selection in chromosomes (d) 12, (e) 15 and (f) 19. Each plot has five tracks that show from top to bottom: genetic differentiation between spring and fall spawners for SNPs with dAF ≥ 0.4; heatmap plot depicting the minor allele frequency per population (rows) for the novel outlier SNPs (columns); average nucleotide diversity (*π*) and Tajima's *D* (window size 10 kbp, step size 2 kbp) for spring and fall spawners, in light and dark blue lines respectively; and estimate of recombination rate (rho/kbp) every 100 kbp (Pettersson et al., 2019). Novel outlier SNPs (dAF ≥ 0.55) are denoted as red filled circles, missense mutations as filled black circles and other SNPs are grey circles. For zoom‐in plots to each peak of divergence including gene names see Figures S8, S9, S11.

In the spring versus fall spawners comparison, we discovered that the most divergent genomic regions were distributed across four chromosomes (*n* = 2137 SNPs, Figure 2a). Further examination of these chromosomes revealed nine ‘islands of divergence’: one on Chr8 (Figure 3), one on Chr12, five on Chr15 and two on Chr19 (Figure 4 and Figure S9). Four of these divergent regions are newly identified as outliers in this study, as they were not among the independent loci reported in Han et al. (2020) (Chr8: 23,040,136–30,729,461, Chr15: 6,750,000–7,000,000, Chr15: 9,200,000–9,350,000, Chr19: 23,155,000–23,300,000). While some SNPs in these regions have been associated with spawning time before (Han et al., 2020) (*n* = 387, blue triangles in Figure 2a), we discovered a large number of SNPs newly identified as outliers (*n* = 1748, 81.8%) (Figures 3 and 4, red dots). Most of these SNPs were located in a 7.7‐Mbp long putative structural variant (SV) on Chr8 (*n* = 1349, 77% of the new outliers) (Figure 3) that has not been reported as such previously. Other outlier SNPs were found in genomic regions with known association with spawning time on Chr12, 15 and 19 (Figure 4).

Our data also revealed that the latitudinal genetic pattern discriminating populations that spawn north or south of a biogeographic transition zone identified on the eastern Scotian Shelf (Figure 1a) is underlied by the large 8‐Mbp long inversion on Chr12 (*n* = 1106 SNPs with dAF ≥ 0.42, Figure 2b) (Pettersson et al., 2019).

### 3.4 An undescribed structural variant on chromosome 8

The newly discovered putative SV distinguishing spring and fall spawners in the NW Atlantic is located towards one end of the chromosome (Figure 3a) in a region with heterogeneous rate of recombination (Figure 3b) (Chr8: 23,040,136–30,729,461). Most outlier SNPs at this locus are undescribed, except for six known spawning time‐ and two salinity‐associated markers (Han et al., 2020) (Figure 3b). This putative SV was not highlighted in Han et al. (2020), but an examination of the patterns of segregation shows that its variant haplotype is present in some populations in the NE Atlantic, though it does not always distinguish spring and fall spawners (Figure S7c).

Alternative haplotypes of the SV differentiate spring and most fall spawners (mean allele frequency in spring spawners = 0.11 ± 0.04, and in fall spawners except NTS‐F = 0.73 ± 0.03), and intermediate allele frequencies were observed in the summer spawning population from Sept Îsles (SIL‐U), the mixed sample from Bras D'Or lake (BDO‐M) and the fall spawning population from Northumberland Strait (NTS‐F) (mean allele frequency of SIL‐U, BDO‐M, NTS‐F = 0.51 ± 0.02) (Figure 3b).

The genetic differentiation at this locus shows the typical profile of a chromosomal inversion, ‘block’ shaped with sharp ‘edges’, which is often interpreted as a result of reduced recombination within the inversion but normal rates outside of it (Figure 3a). An examination of the pattern of LD at this locus based on genotype data of individuals sequenced to a high depth of coverage, confirmed that LD is higher within the SV and lower in flanking regions as expected, however, this pattern appears to be discontinuous, suggesting the possibility of recombination (Figure 3c). To evaluate this, we estimated the extent of allele sharing between the two haplotypes of the SV on Chr8 and compared it with that of the four previously described inversions on Chr6, 12, 17 and 23 (Han et al., 2020). We found that a large proportion of SNPs within the SV on Chr8 are shared between haplotypes, with a stronger enrichment towards one end of the SV, while a smaller proportion of SNPs are diagnostic (not shared between haplotypes, implying restricted recombination) (Figure 3d). This pattern contrasts markedly with that of the four previously reported inversions in Atlantic herring, which overall show a relatively higher enrichment of diagnostic SNPs, from the highest to the lowest in this order: Chr12 > Chr6 > Chr23 > Chr17 > Chr8 (Figure 3d,e).

### 3.5 Newly identified and known signals of selection associated with spawning ecotypes

The selection signal on Chr12 is located upstream of a known inversion (Pettersson et al., 2019) and extends over 1 Mbp towards the middle of the chromosome (Figure 4a) in a region of low recombination (Figure 4d, last track; Figure S8). Notably, most outlier SNPs in this locus are newly described as outliers, except for two spawning time‐associated markers (Figure 4d, first track; Figure S8b, second track) that occur in a region showing strong differentiation in a previous study (Han et al., 2020). Alternative alleles were close to fixation between spring and fall spawners, except in the spring‐spawning sample from Stephenville (SPH‐S, southwest of Newfoundland) that shows intermediate allele frequencies (Figure 4d, second track; Figure S8b, third track). The profile of genetic diversity metrics for the most divergent SNPs revealed that this region likely experienced a selective sweep in the fall spawners, supported by the lower nucleotide diversity and more negative Tajima's *D* than in the spring spawners (Figure 4d, fourth track; Figure S8b, fifth track). Genes in this region include: five genes of the myosin heavy chain family (i.e. *myhc*‐like, *myhz1.1* and three *myh2*), *scd5* (stearoyl‐CoA desaturase 5), *tmem150c* (transmembrane protein 150C), *sbno1* (strawberry Notch Homolog 1) and *denr* (density‐regulated protein). Opposite alleles at this locus distinguish spring and fall spawners in both Canadian and Baltic waters, but this is not applicable to all north Atlantic populations (Figure S8). The Canadian ‘fall’ allele is prevalent in all other oceanic populations (Greenland, Ireland‐UK, North Sea and Norwegian fjords) regardless of their spawning season and in the Baltic fall spawners. There is a gradient of allele frequencies in the transition zone between the Baltic Sea and the NE Atlantic Ocean.

The signal of selection on Chr15 is 4 Mbp long (Figure 4b,e and Figure S9) and it is located towards one end of the chromosome in a region of low recombination. It consists of five distinct loci, two more than detected in the east Atlantic (Han et al., 2020). In genomic order, the first locus is novel, it is approximately 250 kbp long (Chr15: 6,750,000–7,000,000) and it harbours the genes *rab15* (member RAS Oncogene Family 15) and *sipa1l1* (signal induced proliferation associated 1 like 1) (Figure S9c). The second locus extends over 200 kbp (Chr15: 7,650,000–7,850,000) and contains the gene *sox11b* (Figure S9d), which has been associated with spawning time before (Han et al., 2020), but the two most differentiated SNPs are novel (dAF > 0.7), as they did not show consistent association between NE Atlantic and Baltic populations. The third locus is about 530 kbp long (Chr15: 8,540,000–9,070,000) and harbours a large number of SNPs and genes with a known association with spawning time such as *tshr* and *calm1b* (Figure S9e). The nucleotide diversity and Tajima's *D* profiles support a selective sweep in the *tshr* region, in concordance with previous studies (Chen et al., 2021) (Figure S9e). The fourth locus is novel, extends over 150 kbp (Chr15: 9,200,000–9,350,000) and contains two genes, *flrt2* (fibronectin leucine‐rich transmembrane protein 2) and a *lncRNA* (Figure S9f). The fifth locus extends over 280 kbp (Chr15: 10,820,000–11,100,000) and consists of two peaks: one spans two well‐characterized genes, *esr2a* and *syne2* (Figure S9g), and the other one encompasses several *lncRNA* genes and *l3hypdh* (trans‐l‐3‐hydroxyproline dehydratase). Interestingly, most SNPs in the *esr2a*‐*syne2* region are newly described as outliers, while the SNPs in the *lncRNAs*‐*l3hypdh* region have been previously associated with spawning time (Han et al., 2020). Across all five loci in this region, the spring and fall spawners in this study have opposite alleles in high frequency (Figure S9b, second track). When comparing allele frequencies between west and east Atlantic herring, a similar pattern distinguishing spring and fall spawners is observed in three of the five loci, harbouring *tshr*, *sox11b* and *flrt2‐lncRNA* genes and in the second peak of the *esr2a*‐*syne2‐lncRNAs*‐*l3hypdh* locus (*lncRNAs*‐*l3hypdh*) (Figure S10c,e). In the first peak of the latter locus, corresponding to *esr2a*‐*syne2*, the ‘fall’ Canadian allele is predominant among populations from the transition zone and the Baltic Sea (Figure S10c,d). In the *rab15‐sipa1l1* locus, the alternative alleles appear to be unique of Canadian spring spawners (Figure S10a,b).

The selection signal on chromosome 19 is located towards one end of the chromosome, in a region of low recombination, and it contains two loci (Figure 4c,f and Figure S11). The first locus is 410 kbp long (Chr19: 20,290,000–20,700,000) and harbours the genes *herpud2*, *adgrb1b* and a *lncRNA* (Figure S11c). Most SNPs and genes at this locus have been previously associated with spawning time (Han et al., 2020). The second locus is newly described as outlier, extends over 145 kbp (Chr19: 23,155,000–23,300,000) and includes a *lncRNA* and *sgk‐like* genes (Figure S11b). The low nucleotide diversity and negative Tajima's *D* profiles at this region suggest that it constitutes a selective sweep (Figure S11d). Indeed, the variant alleles at this locus are unique for the Canadian fall spawners and the summer‐spawning sample from Greenland (Figure S11e,f). A description of outlier SNPs and the closest genes at divergent genomic regions can be found in Table S4.

### 3.6 The inversion on chromosome 12 is likely associated with minimal temperature tolerance

**FIGURE 5:**
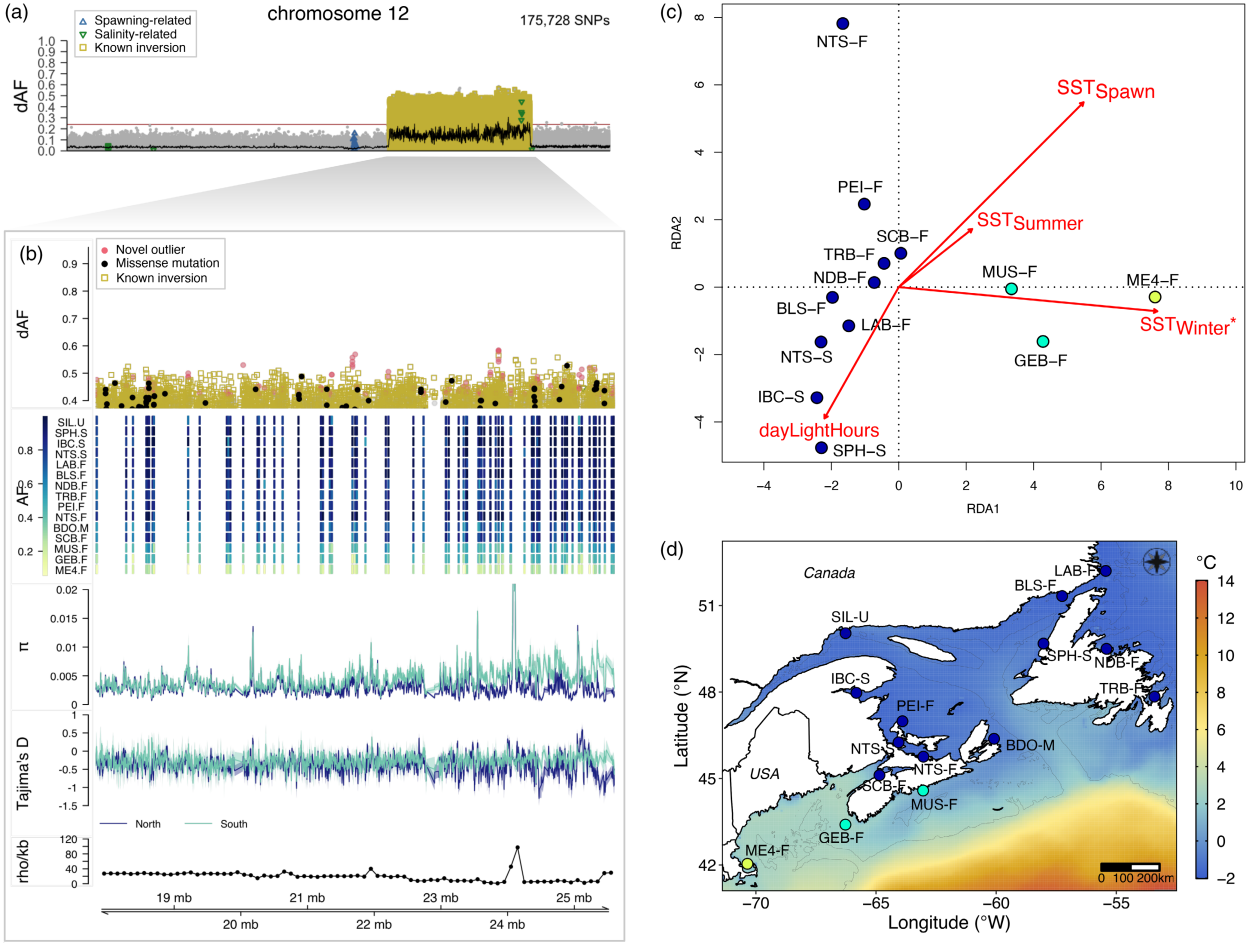
Chromosomal inversion on chromosome 12 associated with spatial genetic divergence along a latitudinal environmental cline. (a) Genetic differentiation (dAF) along the chromosome. (b) Close‐up plot to the putative inversion (Chr12: 17,826,318–25,603,093) consisting of five tracks. The first track is the genetic differentiation for SNPs with dAF ≥ 0.4. Each dot is a single SNP, its shape indicates whether it is a novel outlier (red filled circle), a missense mutation (black circle) or is in one of four inversions reported in Han et al. (2020). The second track depicts the pool‐minor allele frequency of the novel outlier SNPs, where each row is a single pool sample and each column is a SNP. The third and fourth tracks show the profile of the average nucleotide diversity (*π*) and Tajima's *D* of northern and southern populations, calculated in 10 kbp sliding windows with a 2‐kbp step. The fifth track shows the recombination rate (rho/kbp) every 100 kbp (Pettersson et al., 2019). (c) Redundancy analysis plot representing the association between four uncorrelated environmental variables and population allele frequencies of the top outlier SNPs within the inversion on Chr12 (dAF >0.55). The environmental variables are SST_Spawn_: sea surface temperature during spawning, SST_Summer_: sea surface temperature during summer months, SST_Winter_: sea surface temperature during winter months, dayLightHours: Hours or daylight. Each circle corresponds to a spawning aggregation and their colour indicates their predominant population allele frequency. Sample abbreviations and names as in Table 1. The red arrows (and their length) indicate the level of correlation of each environmental variable with genetic variation in the first two axes. The environmental variable with the strongest correlation with allele frequency variation is indicated with an asterisk (*), for an alpha value of statistical significance <0.01. Results of ANOVA to test statistical significance are shown in Table S5. (d) Map depicting average winter sea surface temperature and the predominant population allele frequencies at diagnostic SNPs within the inversion on Chr12 for the 15 spawning aggregations included in this study. Each circle corresponds to a spawning aggregation and their colour indicates their predominant population allele frequency as per in (b) second track.

The inversion on Chr12 that underlies the latitudinal, north–south genetic pattern contains a large number of outlier SNPs as previously reported (Han et al., 2020), but some of the most differentiated ones are newly described as outliers among NW Atlantic populations (Figure 5a,b). The ‘northern’ haplotype of the inversion, as defined in Han et al. (2020), is prevalent among northern samples from Labrador (LAB‐F), Newfoundland (NDB‐F, TRB‐F, SPH‐S), Gulf of St. Lawrence (BLS‐F, SIL‐U, PEI‐F, NTS‐S), Bras D'Or lake (BDO‐M) and Scots Bay in the Bay of Fundy (SCB‐F) (mean allele frequency in northern pools = 0.86 ± 0.02, Figure S12a). The ‘southern’ allele is in high frequency in the sample from the Gulf of Maine (ME4‐F) (allele frequency in ME4‐F = 0.26), the southernmost location included in this study. Intermediate haplotype frequencies were common in two samples from the Scotian Shelf (MUS‐F, GEB‐F) (mean allele frequency MUS‐F and GEB‐F = 0.46 ± 0.04, Figure 5b, second track; Figure S12a).

The current hypothesis is that this putative inversion is likely associated with adaptation to temperature during spawning (Pettersson et al., 2019). To test this hypothesis, we conducted a redundancy analysis (RDA) based on outlier SNPs within the chromosomal inversion on Chr12 (17,823,410–25,605,433) and environmental variables such as the number of hours of day light, average sea surface temperature and salinity during the spawning months (SST_Spawn_) and for the winter (SST_Winter_) and summer (SST_Summer_) seasons, as a proxy for the most extreme annual climatic conditions. This analysis indicated that sea surface winter temperature (SST_Winter_) is the environmental factor that best explains the genetic variation of outlier loci in the Chr12 inversion (*p* = 0.005, *α* = 0.01) (Figure 5c and Figure S13). Indeed, this was the only variable that was statistically significant for an alpha level of 0.01 (Table S5), and salinity measures were not informative (Figure S14). In the RDA plot, spawning aggregations were separated according to SST_Winter_ along RDA 1, axis that explained 58.1% of the total genetic variance. Notably, the ‘north’ haplotype of the inversion is prevalent among populations that seem to be exposed to much colder temperatures during the winter than the southern ones on the Scotian Shelf and the Gulf of Maine (Figure 5d).

## 4 DISCUSSION

Our comprehensive whole‐genome study revealed that genetic differentiation among NW Atlantic herring populations lies primarily in a limited number of highly divergent genomic regions (outlier loci), in otherwise weakly differentiated genomes (mean pairwise $\hat{F}$_ST_ between 0.001 and 0.018) (Figures 1 and 2). This result is in line with prior whole‐genome scans in the species (Han et al., 2020; Lamichhaney et al., 2017; Martinez Barrio et al., 2016). A previous study demonstrated that the $\hat{F}$_ST_ distribution in Atlantic herring deviates significantly from the one expected for selectively neutral alleles under a drift model (Lamichhaney et al., 2017). Thus, we interpret that these major outlier loci may be under natural selection and may contribute to ecological adaptation. In genome scans based on $\hat{F}$_ST_ values, a similar pattern of outlier loci could also occur at regions of reduced recombination, as background selection against deleterious mutations reduces nucleotide diversity within populations (Burri, 2017; Ravinet et al., 2017). However, we consider it unlikely that background selection could explain the outlier loci reported in this study because they were detected using the absolute difference in allele frequency (dAF) between populations, which is an estimate of genetic differentiation that is not affected by background selection as $\hat{F}$_ST_ does. Moreover, the nucleotide diversity (*π*) within populations is not low at these loci (see Figures 3b, 4d–f, 5b). We found 10 highly divergent genomic regions between populations in the NW Atlantic (Table S6). Nine of these regions differentiate populations spawning in different seasons (spring or fall) (Figure 2a) and one distinguishes populations spawning along a latitudinal cline (north or south of a biogeographic transition zone) (Figure 2b). Next, we examine these genomic regions in more detail, discuss the probable evolutionary processes shaping genomic patterns of divergence and the implications of these findings.

### 4.1 Known and undescribed genomic regions distinguishing spawning ecotypes

NW Atlantic herring populations breeding either in spring or fall seasons show striking genetic differences at nine major loci distributed across four chromosomes: 8 (one locus), 12 (one locus), 15 (five loci) and 19 (two loci) (Figures 2a, 3, 4). These loci vary in size, ranging from a few kbp up to several Mbp, as is the case of the 7.7‐Mbp long putative structural variant (SV) on Chr8, first described here for the species (Figure 3). The divergent loci at Chr12, 15 and 19 encompass both, known outlier genetic variants and candidate genes with an association with ecological adaptation in herring (Han et al., 2020), as well as new genetic variants (Figure 4 and Figures S8, S9, S11).

**FIGURE 6:**
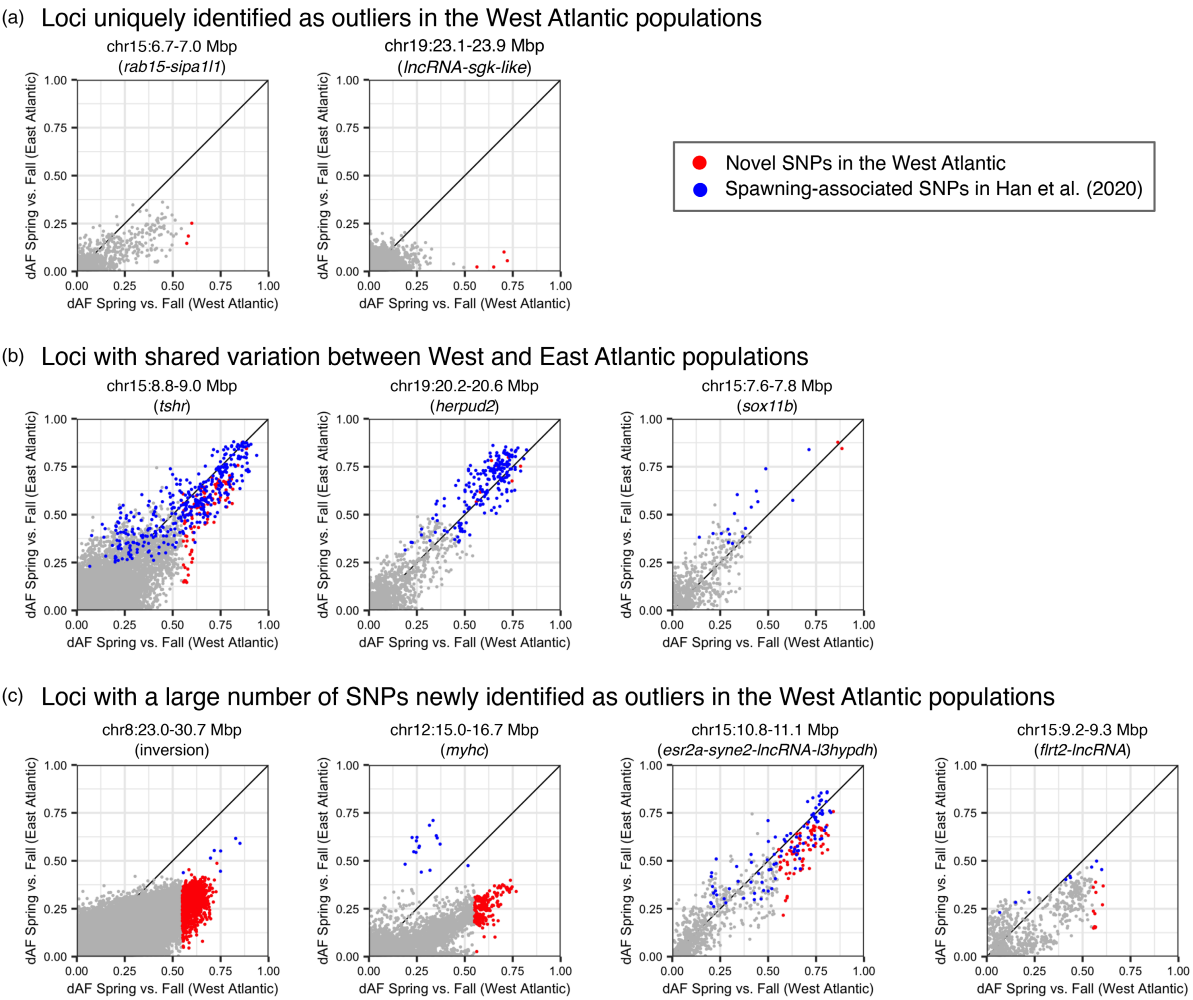
Comparison of the contribution of shared and newly identified outlier SNPs to the genetic differentiation between West and East Atlantic at nine spawning‐associated genomic regions. The absolute difference in allele frequencies (dAF) was used as a measure of genetic differentiation. (a) Loci uniquely identified as outliers in the West Atlantic populations. (b) Loci with shared variation between West and East Atlantic. (c) Loci with a large number of SNPs newly identified as outliers in the West Atlantic populations. Each dot represents a single SNP. Red dots indicate novel SNPs in the West Atlantic and blue dots indicate SNPs with known association with spawning time as reported in Han et al. (2020). The rest of SNPs in the region (not outliers, dAF in the West Atlantic ≤0.55) are shown in grey. A summary of the percentage of unique and shared loci and SNPs is shown in Table S6.

We discovered that the contribution of shared and newly identified outlier SNPs to the differentiation across Atlantic herring populations varies among spawning‐associated genomic regions (Figure 6). Several genetic variants in genes such as *tshr, herpud2, sox11b, esr2a* and *syne2* are shared between NW and NE Atlantic populations (Figure 6b and Figures S9, S11c). Moreover, the alternate haplotypes segregating at these loci and the ranking of the most divergent SNPs are almost identical across populations (Figure 6b and Table S6). This result strongly supports that these genes contribute to seasonal reproduction in Atlantic herring, consistent with previous research (Han et al., 2020; Lamichhaney et al., 2017; Martinez Barrio et al., 2016), added to the fact that several of them have a known function in reproduction in birds and mammals (Bondesson et al., 2015; Melamed et al., 2012; Ono et al., 2008). In contrast, loci with many SNPs newly identified as outliers in the NW Atlantic, show a great diversity in haplotype composition (Figure 6c and Table S6). The best example is the region containing a cluster of myosin heavy chain (*myhc*) genes on Chr12, where numerous SNPs show strong genetic differentiation in NW Atlantic but low differentiation in NE populations, whereas a smaller set of other SNPs show the opposite trend (Figure 6c and Figure S8). In summary, most outlier loci related with spawning seasonality are shared between NW and NE Atlantic populations; however, there is no perfect overlap regarding which SNPs at these loci show the strongest genetic differentiation between populations, as different haplotypes appear to be favoured on each side of the Atlantic.

Some of the candidate genes at spawning‐associated outlier loci have functions that may relate to environmental response and development. For instance, on Chr12, *myhc* in‐tandem gene copies (i.e. *myhc*‐like, *myhz1.1* and *myh2*, Figure 4d and Figure S8) may play an important role in myogenesis and *esr2a* (oestrogen receptor beta) gene (Figure 4e and Figure S9g) is likely essential for female reproduction (Lu et al., 2017). Interestingly, muscle development and oestrogen action are strongly affected by temperature and/or photoperiod (Jin et al., 2010; Johnston et al., 2001), two of the most contrasting environmental conditions between spring and fall seasons. Spring is characterized by colder seawater and increasing day length, whereas fall is characterized by relatively warm seawater and decreasing day length. While selective pressures related to temperature and day length could largely contribute to genetic divergence between herring populations spawning in different seasons, associated factors not included in this study (i.e. salinity, oxygen content, predators, etc.) could also be the underlying cause of selection.

Other outlier genetic variants occur at genes with potentially important roles in embryological development and the regulation of gene expression (Figures S8, S9, S11). For example, on Chr12, *sbno1* (strawberry notch homolog 1) is a gene associated with development of the central nervous system in zebrafish (Takano et al., 2010) and *denr* (density‐regulated protein) is a highly conserved gene involved in translation initiation (Skabkin et al., 2010). On Chr15, *rab15* (member of RAS oncogene family 15) presumably participates in cellular response to insulin stimulus and protein metabolism (Bradford et al., 2022), *sipa1l1* (signal induced proliferation associated 1 like 1) is likely involved in vertebrate embryogenesis (Tsai et al., 2007) and *flrt2* (fibronectin leucine‐rich transmembrane protein 2) regulates embryonic heart morphogenesis in mice (Müller et al., 2011). On Chr19, a long non‐coding RNA (lncRNA) gene may participate in the regulation of gene expression and is unique outlier locus for NW Atlantic populations. Indeed, lncRNA genes are present in several of the genomic regions under selection (Figure 3c,d and Figures S8g,f, S11c,d). Thus, a potentially fruitful avenue for future research could focus on understanding the role of lncRNAs in local adaptation of Atlantic herring and experimental validations of candidate genes are of considerable interest.

### 4.2 A known inversion on chromosome 12 exhibits a latitudinal pattern that correlates with contrasting environmental conditions

NW Atlantic herring populations breeding north or south of a known biogeographic transition zone on the Scotian shelf (approx. 44.61°N) (Stanley et al., 2018), genetically differ at a large locus on Chr12 that corresponds to a known 7.8‐Mbp long chromosomal inversion (Figure 5) (Chr12: 17,823,410‐25,605,433) (Jamsandekar et al., 2023; Pettersson et al., 2019). In our study, spring spawning populations were restricted to the Gulf of St. Lawrence, an area north of the transition zone. We observed that spring and fall spawning populations north of the transition zone have the ‘northern’ haplotype in high frequency; the southernmost population, in Maine (ME4‐F) has predominantly the ‘southern’ haplotype; and intermediate populations south of the transition zone, in Musquodoboit harbor (MUS‐F) and German Banks (GEB‐F), have both haplotypes at intermediate frequencies (Figures 5a and 6b).

While the role of this inversion is unknown, it has been proposed that it is associated with adaptation to temperature during spawning (Pettersson et al., 2019). To assess this, we performed an RDA‐based genome–environment association (GEA) analysis, examining the correlation between the allele frequencies of genetic variants within the inversion and environmental variables at the sampling sites (i.e. the number of hours of day light, average sea surface temperature and salinity during the spawning month and for the winter and summer seasons as representatives of the most extreme climatic conditions experienced annually). GEA indicated that seawater temperature during winter is the most concordant environmental factor with the latitudinal pattern of genomic differentiation shown by the Chr12 inversion (ANOVA *p* ≤ 0.005, *α* = 0.01) (Figure 5c,d and Figures S13, S14; Table S5). It is important to note that here winter temperature refers to the seawater temperature at spawning sites over the winter months, assuming that the first life stages stay close to the spawning site during their first winter. This assumption is supported by previous research indicating that there is persistence of larvae in the vicinity of spawning areas during the first 2–3 months after the breeding season (Sinclair & Power, 2015). The RDA was driven by 3 of the 13 populations studied (Figure 5c), and the population that is the main driver of this result (ME4‐F) is the southernmost population. The steep thermal gradient in the area suggests that during the winter herring larvae present in the north of the transition zone may experience much colder temperatures on average than those in the south (Figure 5d). Temperature has a particularly strong influence on the physiology of early life stages of marine species which, due to physiological constrains and high mortality during this phase, are particularly sensitive to thermal conditions (Dahlke et al., 2020; Marr, 1956). Indeed, temperature has been shown to also have a strong influence on the epigenome of Atlantic herring larvae by affecting DNA methylation patterns (Kho et al., 2023). Thus, temperature, or associated factors, may constitute an important selective pressure for herring larvae. Notably, the latitudinal genetic pattern observed in NW Atlantic herring agrees with similar studies conducted in the same area for a number of other marine species [i.e. Atlantic cod (*Gadus morhua*), American lobster (*Homarus americanus*), sea scallop (*Placopecten magellanicus*), northern shrimp (*Pandalus borealis*) and the invasive European green crab (*Carcinus maenas*)] (Stanley et al., 2018). Consequently, our results suggest that climate‐related selective pressures affecting early‐life survival during the winter months (i.e. post‐settlement mortality) may have shaped the latitudinal genetic pattern observed in herring. Connectivity during early life stages of herring in the NW Atlantic has been generally assumed to be limited, corresponding to fine spatial scales of population structure (Stephenson et al., 2009). Our results correspond to and further illustrate this hypothesis, whereby emergent genetic patterns associated with adult fish correspond to processes likely imparting selection on earlier life phases.

The prevalence of intermediate allele frequencies near the transition zone suggests that climatic conditions may vary between years in relation to oceanographic regional trends (Townsend et al., 2004). In a study of genetic structure of the invasive European green crab (*Carcinus maenas*), Jeffery et al. (2018) similarly identified intermediate populations proximate to the biogeographic break within our focal area. It is possible that populations at these locations experience significant inter‐annual environmental fluctuations during winter, depending on the strength of the warm Gulf Stream flowing north or of the cold Labrador Current flowing south. Thus, it is possible that balancing selection may be favouring both inversion haplotypes at locations near the transition zone.

In addition to natural selection, demographic history or secondary contact of divergent lineages may result in a latitudinal genetic pattern similar to that observed here. Under these alternate scenarios, the expectation is that both outlier and neutral loci would exhibit a similar latitudinal pattern, as observed between two invasive European green crab (*Carcinus maenas*) lineages that got in contact after being introduced to eastern North America (Jeffery et al., 2018). This is not the case for herring, where the latitudinal pattern is only observed at outlier loci, whereas genome‐wide divergence is minimal. However, we cannot exclude that outlier loci originated from divergent lineages and that differences in allele frequencies at these loci have been maintained by natural selection, while differences in allele frequencies at neutral loci have been erased after secondary contact (Le Moan et al., 2016; Ravinet et al., 2017; Rougeux et al., 2017).

### 4.3 The structural variant on chromosome 8 has presumably high gene flux

Another interesting finding from this study is that the undescribed spawning‐associated SV on Chr8 (Figure 3a–c), exhibits an unexpectedly high degree of allele exchange between haplotypes (gene flux) compared to the four confirmed inversions on Chr6, 12, 17 and 23 (Jamsandekar et al., 2023) (Figure 3d,e). Our results suggest that this SV has the highest gene flux of all large SVs reported in Atlantic herring to date [from highest to lowest gene flux (size in Mb shown within parenthesis): SV on Chr8 (7.7 Mb) > Chr17 (1.8 Mb) > Chr23 (1.4 Mb) > Chr6 (2.6 Mb) > Chr12 (7.8 Mb) (Figure 3d,e)]. Additionally, this SV shows an unexpected heterogeneous profile of divergence, including ‘peaks’ or regions within the SV with elevated genetic differentiation (e.g. cluster of spawning‐related SNPs shown as blue triangles in Figure 3b), which deviate from the expected pattern of a canonical inversion with suppressed recombination.

While size, age and composition may influence the level of gene flux and recombination of chromosomal rearrangements, in the absence of additional data we find it challenging to infer what processes may have led to the pattern observed in this SV. Chromosomal rearrangements such as inversions impact genetic divergence, adaptation and speciation by reducing recombination in heterokaryotypes (Berdan et al., 2023; Hoffmann & Rieseberg, 2008; Wellenreuther & Bernatchez, 2018). Double crossovers and loop pairings between rearranged chromosome portions are more likely to occur in larger inversions than in smaller inversions. However, in herring, size differences do not appear to explain the variation in gene flux among SVs, as the inversion on Chr12 is similar in size to the SV on Chr8, but has been less affected by gene flux. Older inversions might be more prone to be eroded than younger ones, as they have had time to accumulate gene conversion and double crossover events. The frequency of heterokaryotypes also affects the number of recombination events, since an inversion with numerous heterokaryotypes tends to have more gene flux than inversions that primarily occur as homokaryotypes. One possible explanation for the narrow divergence peaks observed within the inversion is the presence of small nested structural variants, as seen in the yellow monkey flower (Kollar et al., 2023). However, this pattern could also result from methodological artefacts related to segmental duplications and gene conversions that might bias genome assembly in the region and mapping of pool‐seq short reads between divergent genomic regions. Therefore, the generation of long‐read sequence data is necessary to examine these hypotheses.

### 4.4 Genetically differentiated spawning ecotypes with incomplete reproductive isolation

Speciation is the development of reproductive isolation among populations leading to new species. This complex and continuous process involves the existence of barriers to gene flow that can affect the genome differently. When gene flow takes place during the speciation process, barrier loci that restrict gene flow appear as ‘peaks’ or regions of elevated genetic differentiation that stand out from a ‘shallow’ undifferentiated genomic background homogenized via gene flow. This pattern is often referred to as ‘heterogeneous landscape of differentiation’ (Ravinet et al., 2017). In the case of Atlantic herring, we observe such genomic heterogeneity between spawning ecotypes (Han et al., 2020). Indeed, the 120 kb *tshr* locus has the strongest association with reproductive seasonality, as it shows substantial genetic differences between spring‐ and autumn‐spawning herring across the North Atlantic and Baltic Sea (Chen et al., 2021; Lamichhaney et al., 2017). Differences in breeding time (reproductive allochrony) can potentially constitute barriers to gene flow leading to reproductive isolation and population divergence (Taylor & Friesen, 2017). This could explain the significant genetic differences observed along the *tshr* locus and suggest that spawning time may be determined by an important genetic component interacting with the environment. However, previous studies show that spawning time is not strictly genetically determined as some plasticity occurs (Berg et al., 2021; Folkvord et al., 2009; Han et al., 2020). For instance, Han et al. (2020) documented a population spawning in the autumn that was classified as spring‐spawning herring based on both genetic and otolith data. This and other observations suggest that gene flow can take place between spawning ecotypes, which explains the lack of genetic differentiation at neutral loci. Taking these observations into account, we suggest it is most appropriate to consider spring‐ and autumn‐spawning herring as different ecotypes with incomplete reproductive isolation, though it is possible that this could lead to speciation in the future.

### 4.5 Implications

Our work contributes to our understanding of the evolutionary biology of Atlantic herring and has implications for the management of the species and, perhaps for that, of other marine species with comparable life histories. This study expands our understanding of the genomic basis of population divergence and adaptation with gene flow by: (i) contributing a list of candidate genetic variants and genes likely associated with intra‐specific diversity; (ii) providing genetic evidence supporting the hypothesis that strong selection can maintain molecular divergence at loci underlying variation of phenotypic traits and fitness in large populations despite the presence of gene flow; (iii) illustrating how such selection can be driven by ecological and environmental factors varying at different temporal and spatial scales and (iv) adding to the growing body of evidence for the central role of structural variants in adaptation with gene flow. This study also suggests the complexity of predicting how climate change will influence marine populations that exhibit fine‐scale, environmentally mediated population structure, whereby changes in temperature could elicit asymmetric responses among population subgroups and potentially disrupting synchrony between reproductive timing, larval emergence and seasonal prey availability [sensu (Cushing, 1990) – ‘Match–Mismatch hypothesis’]. Our results are consistent with previous research indicating that genetic adaptation in Atlantic herring is to a large extent based on ancestral haplotype blocks, some associated with inversions, that have persisted over hundreds of thousand years (Han et al., 2020; Lamichhaney et al., 2017; Pettersson et al., 2019). However, these haplotype blocks are not static over time, they continue to accumulate causal changes contributing to genetic adaptation, as observed in this study. This is a mode of evolution also noted in the Darwin's finches and in other adaptive radiations (Rubin et al., 2022). Taken together, this work contributes to the growing body of evidence supporting the hypothesis that population structure in marine species is often adaptive and underlies ecological diversity (Benestan et al., 2015; Bradbury et al., 2013; Han et al., 2020; Kess et al., 2021; Lehnert et al., 2018).

## CONFLICT OF INTEREST STATEMENT

The authors declare no competing interest.

## Supporting information


Appendix S1.

Table S4.

## Data Availability

Both individual and pooled DNA sequence data generated in this study are deposited in the Sequence Read Archive (SRA) of the National Center for Biotechnology Information (NCBI) under the BioProject PRJNA930418. The reference allele frequencies and environmental data associated to 15 pools of NW Atlantic Herring (*Clupea harengus*) are available in DRYAD repository under the DOI: 10.5061/dryad.0rxwdbs6k. Custom scripts are available in the Github repository: https://github.com/apfuentes/2024_NWAtlanticHerring_PopGen.
