# Supplementary material for: Adaptation to seasonal reproduction and environment‐associated factors drive temporal and spatial differentiation in northwest Atlantic herring despite gene flow
Source: Evol Appl. 2024 Mar 14;17(3):e13675. doi: 10.1111/eva.13675 (PMC10940790; doi:10.1111/eva.13675)
Supplement: Supplementary file 1 — Appendix S1. [file EVA-17-e13675-s002.docx]

**Supporting Information for:**

**Adaptation to seasonal reproduction and environment-associated factors drives temporal and spatial differentiation in northwest Atlantic herring despite gene flow**

**Table of Contents:**

| **Table S1** | Page 2 |
| --- | --- |
| **Table S2** | Page 3 |
| **Table S3** | Page 4 |
| **Table S4** | Page 4 |
| **Table S5** | Page 4-5 |
| **Table S6** | Page 6 |
| **Figure S1** | Page 7 |
| **Figure S2** | Page 8 |
| **Figure S3** | Page 9 |
| **Figure S4** | Page 10 |
| **Figure S5** | Page 11 |
| **Figure S6** | Page 12 |
| **Figure S7** | Page 13 |
| **Figure S8** | Page 14 |
| **Figure S9** | Page 16 |
| **Figure S10** | Page 18 |
| **Figure S11** | Page 19 |
| **Figure S12** | Page 21 |
| **Figure S13** | Page 22 |
| **Figure S14** | Page 22 |
| **Supplementary references** | Page 23 |

**Table S1.** Pool data samples used from Han et al (2020), of which 47 correspond to Atlantic herring and one to Pacific herring.

| **Pool data** | | | | | | | | |
| --- | --- | --- | --- | --- | --- | --- | --- | --- |
| **Sample name** | **Latitude** | **Longitude** | **Region** | **Spawning season** | **Sampling date** | **Sample size** | **Salinity (ppt)** | **Reference** |
| A_Kalix_Baltic_Spring | 65.52 | 22.43 | Baltic Sea | Spring | 19800629 | 47 | 3 | Lamichhaney et al., 2012 |
| HGS1_Riga_Baltic_Spring | 58.34 | 24.62 | Baltic Sea | Spring | 20140421 | 96 | 5.5 | Hill et al. 2019; Bekkevold et al. 2016 |
| HGS2_Riga_Baltic_Spring | 58.34 | 24.62 | Baltic Sea | Spring | 20160530 | 100 | 5.5 | Hill et al. 2019; Bekkevold et al. 2016 |
| HGS3_Riga_Baltic_Autumn | 58.10 | 23.92 | Baltic Sea | Autumn | 20140903 | 96 | 5.5 | Hill et al. 2019; Bekkevold et al. 2016 |
| HGS4_Riga_Baltic_Autumn | 58.10 | 23.92 | Baltic Sea | Autumn | 20150909 | 99 | 5.5 | Hill et al. 2019; Bekkevold et al. 2016 |
| PB6_Gävle_Baltic_Summer | 60.43 | 17.18 | Baltic Sea | Summer | 20120718 | 100 | 6 | Martinez Barrio et al. 2016 |
| B_Vaxholm_Baltic_Spring | 59.26 | 18.18 | Baltic Sea | Spring | 19790827 | 50 | 6 | Lamichhaney et al. 2012 |
| PB1_Hästskär_Baltic_Spring | 60.35 | 17.48 | Baltic Sea | Spring | 20130522 | 50 | 6 | Martinez Barrio et al. 2016 |
| PB4_Hudiksvall_Baltic_Spring | 61.45 | 17.30 | Baltic Sea | Spring | 20120419 | 100 | 6 | Martinez Barrio et al. 2016 |
| PB5_Gävle_Baltic_Spring | 60.43 | 17.18 | Baltic Sea | Spring | 20120507 | 100 | 6 | Martinez Barrio et al. 2016 |
| PB7_Gävle_Baltic_Autumn | 60.44 | 17.35 | Baltic Sea | Autumn | 20120904 | 100 | 6 | Martinez Barrio et al. 2016 |
| G_Gamleby_Baltic_Spring | 57.50 | 16.27 | Baltic Sea | Spring | 19790820 | 49 | 7 | Lamichhaney et al. 2012 |
| PB11_Kalmar_Baltic_Spring | 57.39 | 17.07 | Baltic Sea | Spring | 20120509 | 100 | 7 | Martinez Barrio et al. 2016 |
| PB12_Karlskrona_Baltic_Spring | 56.10 | 15.33 | Baltic Sea | Spring | 20120530 | 100 | 7 | Martinez Barrio et al. 2016 |
| HGS71_Rugen_Baltic_Spring | 54.14 | 13.47 | Baltic Sea - Transision | Spring | 20090406 | 40 | 8 | Hill et al. 2019; Limborg et al. 2012 |
| HGS72_Rugen_Baltic_Spring | 54.14 | 13.47 | Baltic Sea - Transision | Spring | 20030424 | 40 | 8 | Hill et al. 2019; Limborg et al. 2012 |
| PN3_CentralBaltic_Baltic_Spring | 55.24 | 15.51 | Baltic Sea (=Transition gene pool) | Spring | 20111018 | 100 | 8 | Martinez Barrio et al. 2016 |
| S18_Germany_Baltic | 54.22 | 13.58 | Baltic Sea - Transision | Spring | 20120320 | 110 | 8 | Present study |
| HGS12_BornholmBasin_Baltic_Autumn | 55.30 | 15.22 | Baltic Sea - Transision | Autumn | 20161110 | 45 | 8 | Hill et al. 2019 |
| HGS6_Schlei_Baltic_Spring | 54.60 | 9.76 | Baltic Sea - Transision | Spring | 20110509 | 50 | 9 | Hill et al. 2019; Bekkevold et al. 2015 |
| HGS5_Schlei_Baltic_Autumn | 54.60 | 9.76 | Baltic Sea - Transision | Autumn | 20101025 | 89 | 9 | Hill et al. 2019 |
| H_Fehmarn_Baltic_Autumn | 54.50 | 11.30 | Baltic Sea - Transision | Autumn | 19790923 | 50 | 12 | Lamichhaney et al. 2012 |
| HGS11_RingkobingFjord_Spring | 56.02 | 8.19 | Northeast Atlantic Ocean - Transition | Spring | 20090422 | 40 | 12 | Hill et al. 2019; Limborg et al. 2012 |
| HGS24_Landvik_Atlantic_Spring | 58.32 | 8.50 | Northeast Atlantic Ocean - Transition | Spring | 20150428 | 30 | 15 | Present study |
| HGS24_Landvik_Atlantic_Spring | 58.32 | 8.50 | Northeast Atlantic Ocean - Transition | Spring | 20150506 | 16 | 15 | Present study |
| HGS24_Landvik_Atlantic_Spring | 58.32 | 8.50 | Northeast Atlantic Ocean - Transition | Spring | 20150603 | 4 | 15 | Present study |
| LandvikS17_Norway_Atlantic_Spring | 58.32 | 8.50 | Northeast Atlantic Ocean - Transition | Spring | 20150519 | 38 | 15 | Present study |
| J_Träslövslage_Baltic_Spring | 57.03 | 12.11 | Northeast Atlantic Ocean - Transition | Spring | 19781023 | 50 | 20 | Lamichhaney et al. 2012 |
| PB9_Kattegat_Atlantic_Spring | 57.43 | 11.42 | Northeast Atlantic Ocean - Transition | Spring | 20120312 | 100 | 23 | Martinez Barrio et al. 2016 |
| O_Hamburgsund_Atlantic_Spring | 58.30 | 11.13 | Northeast Atlantic Ocean - Transition | Spring | 19790319 | 49 | 25 | Lamichhaney et al. 2012 |
| HGS8_KattegatNorth_Atlantic_Spring | 57.40 | 11.40 | Northeast Atlantic Ocean - Transition | Spring | 20090424 | 41 | 25 | Hill et al. 2019; Limborg et al. 2012 |
| PB10_Skagerrak_Atlantic_Spring | 58.19 | 11.21 | Northeast Atlantic Ocean - Transition | Spring | 20120320 | 100 | 25 | Martinez Barrio et al. 2016 |
| HGS25_Lindås_Atlantic_Spring | 60.73 | 5.13 | Northeast Atlantic Ocean | Spring | 20100312 | 50 | 28 | Present study |
| HGS26_Lusterfjorden_Atlantic_Spring | 61.48 | 7.58 | Northeast Atlantic Ocean | Spring | 20111108 | 50 | 32 | Present study |
| HGS23_Clyde_Atlantic_Spring | 55.14 | -5.04 | Northeast Atlantic Ocean | Spring | 20030314 | 39 | 33 | Present study; Hatfield et al., 2007 |
| HGS17_IsleOfMan_IrishSea_Autumn | 54.06 | -4.37 | Northeast Atlantic Ocean | Autumn | 20150930 | 50 | 33 | Present study |
| HGS19_TeelinBay_Atlantic_Winter | 54.63 | -8.63 | Northeast Atlantic Ocean | Winter | 20160108 | 47 | 34 | Present study |
| HGS10_Downs_EnglishChannel_Winter | 51.34 | 1.90 | Northeast Atlantic Ocean | Winter | 20161212 | 55 | 35 | Present study |
| HGS9_Greenland_Atlantic_Spring | 60.78 | -47.15 | Northwest Atlantic Ocean | Summer | 20161005 | 38 | 35 | Present study |
| HGS15_NSSH_Atlantic_Spring | 67.46 | 9.47 | Northeast Atlantic Ocean | Spring | 20170220 | 43 | 35 | Hill et al. 2019 |
| HGS20_Skye_Atlantic_Spring | 57.41 | -6.13 | Northeast Atlantic Ocean | Spring | 20040224 | 50 | 35 | Present study; Hatfield et al., 2007 |
| HGS27_Gloppen_Atlantic_Spring | 61.77 | 6.16 | Northeast Atlantic Ocean | Spring | 20100802 | 20 | 35 | Present study |
| HGS27_Gloppen_Atlantic_Spring | 61.77 | 6.16 | Northeast Atlantic Ocean | Spring | 20120401 | 11 | 35 | Present study |
| HGS27_Gloppen_Atlantic_Spring | 61.77 | 6.16 | Northeast Atlantic Ocean | Spring | 20120905 | 15 | 35 | Present study |
| HGS27_Gloppen_Atlantic_Spring | 61.77 | 6.16 | Northeast Atlantic Ocean | Spring | 20121130 | 4 | 35 | Present study |
| Q_Norway_Atlantic_Atlantic_Spring | 64.52 | 10.15 | Northeast Atlantic Ocean | Spring | 19800207 | 49 | 35 | Lamichhaney et al. 2012 |
| PB2_Iceland_Atlantic_Spring | 65.49 | -12.58 | Northeast Atlantic Ocean | Spring | 20110915 | 100 | 35 | Martinez Barrio et al. 2016 |
| HGS21_Hebrides_Atlantic_Mixed | 58.17 | -7.23 | Northeast Atlantic Ocean | Mixed | 20160828 | 50 | 35 | Present study |
| HGS18_CelticSea_Atlantic_AutumnWinter | 51.59 | -6.51 | Northeast Atlantic Ocean | Winter | 20151201 | 50 | 35 | Present study |
| HGS16_Orkney_NorthSea_Autumn | 59.00 | -2.00 | Northeast Atlantic Ocean | Autumn | 20150901 | 49 | 35 | Present study |
| HGS22_CapeWrath_Atlantic_Autumn | 58.61 | -4.37 | Northeast Atlantic Ocean | Autumn | 20150910 | 49 | 35 | Present study |
| N_NorthSea_Atlantic_Autumn | 58.06 | 6.10 | Northeast Atlantic Ocean | Autumn | 19790805 | 49 | 35 | Lamichhaney et al. 2012 |
| Pacific herring |  |  | Pacific Ocean | Spring | 20121124 | 50 | 35 | Martinez Barrio et al. 2016 |

**Table S2.** Read mapping summary statistics of the pool and individual sequence data included in this study.

| **Locality** | **Code** | **Total reads (millions)** | **GC (%)** | **Insert size (in bp)** | **Median coverage (X)** | **Mean coverage (X)** |
| --- | --- | --- | --- | --- | --- | --- |
| Sept Îles | SIL-U | 478.9 | 43 | 546 | 66.0 | 20.6 |
| Inner Baie Des Chaleurs | IBC-S | 434.5 | 43 | 478 | 57.0 | 17.7 |
| Stephenville | SPH-S | 510.3 | 43 | 505 | 72.0 | 22.8 |
| Northumberland Strait | NTS-S | 426.6 | 44 | 523 | 58.0 | 19.3 |
| Northumberland Strait | NTS-F | 431.9 | 43 | 504 | 57.0 | 17.7 |
| Labrador | LAB-F | 474.7 | 43 | 534 | 69.0 | 22.4 |
| Blanc Sablon | BLS-F | 487.9 | 43 | 531 | 67.0 | 20.6 |
| Notre Dame Bay | NDB-F | 472.4 | 43 | 534 | 69.0 | 22.5 |
| Trinity Bay | TRB-F | 453.5 | 43 | 503 | 63.0 | 20.0 |
| Prince Edward Island | PEI-F | 479.7 | 43 | 536 | 69.0 | 22.5 |
| Bras D’Or lake | BDO-M | 488.9 | 43 | 526 | 68.0 | 21.7 |
| Scots Bays | SCB-F | 502.5 | 43 | 539 | 73.0 | 23.5 |
| Musquodoboit | MUS-F | 470.7 | 43 | 533 | 68.0 | 21.6 |
| German Banks | GEB-F | 424.8 | 43 | 532 | 57.0 | 17.8 |
| Maine fishing area 514 | ME4-F | 558.8 | 44 | 469 | 77.0 | 26.9 |
| Blanc Sablon | 144Sbs344_Canada_Atlantic_Autumn | 227637582 | 44 | 323 | 39.0 | 13.6 |
| Blanc Sablon | 144Sbs349_Canada_Atlantic_Autumn | 213936914 | 44 | 320 | 37.0 | 13.3 |
| Prince Edward Island | 14F4TL404_Canada_Atlantic_Autumn | 247077100 | 44 | 335 | 42.0 | 14.3 |
| Prince Edward Island | 14F4TL415_Canada_Atlantic_Autumn | 186700351 | 44 | 337 | 32.0 | 11.5 |
| Musquodoboit | 14F4WK306_Canada_Atlantic_Autumn | 206257251 | 44 | 340 | 35.0 | 11.9 |
| Musquodoboit | 14F4WK316_Canada_Atlantic_Autumn | 214114883 | 44 | 339 | 36.0 | 12.1 |
| Labrador | 15F2J602_Canada_Atlantic_Autumn | 229188843 | 44 | 333 | 39.0 | 13.4 |
| Labrador | 15F2J606_Canada_Atlantic_Autumn | 261861781 | 44 | 332 | 45.0 | 15.9 |
| Labrador | 15F2J603_Canada_Atlantic_Autumn | 229530928 | 44 | 325 | 39.0 | 13.2 |
| Labrador | 15F2J616_Canada_Atlantic_Autumn | 226727274 | 44 | 326 | 39.0 | 13.6 |
| Notre Dame Bay | 15F3K609_Canada_Atlantic_Autumn | 264031196 | 44 | 331 | 45.0 | 15.2 |
| Notre Dame Bay | 15F3K621_Canada_Atlantic_Autumn | 184647323 | 44 | 329 | 31.0 | 10.4 |
| Notre Dame Bay | 15F3K601_Canada_Atlantic_Autumn | 195462243 | 44 | 334 | 33.0 | 11.1 |
| Notre Dame Bay | 15F3K602_Canada_Atlantic_Autumn | 265286170 | 44 | 336 | 46.0 | 16.2 |
| Scots Bays | 15F4XRsb625_Canada_Atlantic_Autumn | 227597338 | 44 | 340 | 39.0 | 13.5 |
| Scots Bays | 15F4XRsb626_Canada_Atlantic_Autumn | 270358384 | 44 | 332 | 47.0 | 16.7 |
| Gulf of Maine | 15F5Y514-617_US_Atlantic_Autumn | 233585189 | 45 | 311 | 39.0 | 13.1 |
| Gulf of Maine | 15F5Y514-620_US_Atlantic_Autumn | 210847164 | 45 | 322 | 35.0 | 11.9 |
| Bonavista Bay | F3L312_Canada_Atlantic_Autumn | 402639160 | 44 | 349 | 62.0 | 21.0 |
| Bonavista Bay | F3L337_Canada_Atlantic_Autumn | 360420682 | 44 | 329 | 63.0 | 20.8 |
| Northumberland Strait | F4TH327_Canada_Atlantic_Autumn | 370103936 | 44 | 346 | 60.0 | 19.7 |
| Northumberland Strait | F4TH337_Canada_Atlantic_Autumn | 501561278 | 45 | 349 | 31.0 | 10.1 |
| German Banks | F4XQgb402_Canada_Atlantic_Autumn | 414687491 | 45 | 324 | 72.0 | 25.1 |
| German Banks | F4XQgb408_Canada_Atlantic_Autumn | 237496563 | 45 | 340 | 40.0 | 13.8 |
| Stephenville | 12S4Rsv38_Canada_Atlantic_Spring | 240271559 | 45 | 327 | 41.0 | 14.4 |
| Stephenville | 12S4Rsv41_Canada_Atlantic_Spring | 242329251 | 45 | 311 | 42.0 | 14.8 |
| Fortune Bay | 14S3Ps233_Canada_Atlantic_Spring | 245506541 | 44 | 337 | 42.0 | 14.6 |
| Fortune Bay | 14S3Ps265_Canada_Atlantic_Spring | 224401221 | 44 | 336 | 39.0 | 13.7 |
| Notre Dame Bay | 15S3K404_Canada_Atlantic_Spring | 240960201 | 44 | 319 | 41.0 | 13.8 |
| Notre Dame Bay | 15S3K449_Canada_Atlantic_Spring | 212699059 | 44 | 331 | 36.0 | 12.2 |
| Notre Dame Bay | 15S3K410_Canada_Atlantic_Spring | 234394601 | 44 | 328 | 40.0 | 13.8 |
| Notre Dame Bay | 15S3K430_Canada_Atlantic_Spring | 226781013 | 44 | 325 | 39.0 | 13.9 |
| Placentia Bay | 16S4Pla78_Canada_Atlantic_Spring | 239105010 | 44 | 343 | 41.0 | 14.2 |
| Placentia Bay | 16S4Pla79_Canada_Atlantic_Spring | 213310086 | 44 | 336 | 36.0 | 12.2 |
| Placentia Bay | 16S6Pla71_Canada_Atlantic_Spring | 233698231 | 45 | 340 | 39.0 | 13.2 |
| Placentia Bay | 16S6Pla72_Canada_Atlantic_Spring | 236969622 | 44 | 333 | 41.0 | 14.7 |
| Bras D’Or lake | 16SBDO106_Canada_Atlantic_Spring | 280966226 | 44 | 334 | 49.0 | 17.0 |
| Bras D’Or lake | 16SBDO107_Canada_Atlantic_Spring | 201596868 | 44 | 329 | 34.0 | 11.5 |
| Fortune Bay | S3Ps246_Canada_Atlantic_Spring | 300788412 | 44 | 341 | 52.0 | 17.9 |
| Northumberland Strait | S4TH231_Canada_Atlantic_Spring | 338701955 | 44 | 312 | 59.0 | 20.8 |
| Northumberland Strait | S4TH244_Canada_Atlantic_Spring | 231353225 | 45 | 336 | 39.0 | 13.3 |
| Inner Baie Des Chaleurs | S4TM205_Canada_Atlantic_Spring | 289610955 | 45 | 316 | 48.0 | 17.3 |
| Inner Baie Des Chaleurs | S4TM211_Canada_Atlantic_Spring | 257850195 | 45 | 332 | 43.0 | 14.7 |
| Seven Islands | 12S4S7i22_Canada_Atlantic_Summer | 224724100 | 44 | 341 | 38.0 | 12.7 |
| Seven Islands | 12S4S7i28_Canada_Atlantic_Summer | 239760538 | 44 | 337 | 41.0 | 14.3 |

**Table S3.** Pairwise $\hat{F}_{ST}^{pool}$ for 15 herring spawning aggregations in the northwest Atlantic.

|  | **BDO-M** | **BLS-F** | **GEB-F** | **IBC-S** | **LAB-F** | **ME4-F** | **PEI-F** | **MUS-F** | **NDB-F** | **NTS-F** | **NTS-S** | **SCB-F** | **SIL-U** | **SPH-S** | **TRB-F** |
| --- | --- | --- | --- | --- | --- | --- | --- | --- | --- | --- | --- | --- | --- | --- | --- |
| **BDO-M** | 0 | 0.0022 | 0.0024 | 0.006 | 0.0011 | 0.0065 | 0.0015 | 0.001 | 9.00E-04 | 0.0026 | 0.004 | 0.0022 | 0.0062 | 0.0077 | 0.0015 |
| **BLS-F** | 0.0022 | 0 | 0.0037 | 0.0098 | 0.0012 | 0.0086 | 0.002 | 0.0022 | 0.0013 | 0.0035 | 0.0077 | 0.0021 | 0.0077 | 0.0105 | 0.0021 |
| **GEB-F** | 0.0024 | 0.0037 | 0 | 0.0114 | 0.0024 | 0.0049 | 0.003 | 0.001 | 0.0022 | 0.0047 | 0.0094 | 0.0024 | 0.009 | 0.0124 | 0.0026 |
| **IBC-S** | 0.006 | 0.0098 | 0.0114 | 0 | 0.0088 | 0.0164 | 0.0087 | 0.0098 | 0.0083 | 0.0086 | 0.0032 | 0.0113 | 0.0108 | 0.0085 | 0.0087 |
| **LAB-F** | 0.0011 | 0.0012 | 0.0024 | 0.0088 | 0 | 0.0073 | 0.001 | 0.001 | 3.00E-04 | 0.0026 | 0.0068 | 0.001 | 0.0069 | 0.0097 | 0.001 |
| **ME4-F** | 0.0065 | 0.0086 | 0.0049 | 0.0164 | 0.0073 | 0 | 0.0077 | 0.0043 | 0.0068 | 0.0094 | 0.0145 | 0.0068 | 0.0139 | 0.0175 | 0.0072 |
| **PEI-F** | 0.0015 | 0.002 | 0.003 | 0.0087 | 0.001 | 0.0077 | 0 | 0.0016 | 0.001 | 0.0029 | 0.0067 | 0.0018 | 0.0072 | 0.0096 | 0.0016 |
| **MUS-F** | 0.001 | 0.0022 | 0.001 | 0.0098 | 0.001 | 0.0043 | 0.0016 | 0 | 8.00E-04 | 0.0032 | 0.0077 | 0.0013 | 0.0075 | 0.0106 | 0.0014 |
| **NDB-F** | 9.00E-04 | 0.0013 | 0.0022 | 0.0083 | 3.00E-04 | 0.0068 | 0.001 | 8.00E-04 | 0 | 0.0023 | 0.0063 | 0.0012 | 0.0067 | 0.0092 | 0.001 |
| **NTS-F** | 0.0026 | 0.0035 | 0.0047 | 0.0086 | 0.0026 | 0.0094 | 0.0029 | 0.0032 | 0.0023 | 0 | 0.0064 | 0.004 | 0.0081 | 0.0098 | 0.0029 |
| **NTS-S** | 0.004 | 0.0077 | 0.0094 | 0.0032 | 0.0068 | 0.0145 | 0.0067 | 0.0077 | 0.0063 | 0.0064 | 0 | 0.0092 | 0.0089 | 0.0066 | 0.0068 |
| **SCB-F** | 0.0022 | 0.0021 | 0.0024 | 0.0113 | 0.001 | 0.0068 | 0.0018 | 0.0013 | 0.0012 | 0.004 | 0.0092 | 0 | 0.0083 | 0.0118 | 0.0019 |
| **SIL-U** | 0.0062 | 0.0077 | 0.009 | 0.0108 | 0.0069 | 0.0139 | 0.0072 | 0.0075 | 0.0067 | 0.0081 | 0.0089 | 0.0083 | 0 | 0.0125 | 0.0074 |
| **SPH-S** | 0.0077 | 0.0105 | 0.0124 | 0.0085 | 0.0097 | 0.0175 | 0.0096 | 0.0106 | 0.0092 | 0.0098 | 0.0066 | 0.0118 | 0.0125 | 0 | 0.0098 |
| **TRB-F** | 0.0015 | 0.0021 | 0.0026 | 0.0087 | 0.001 | 0.0072 | 0.0016 | 0.0014 | 0.001 | 0.0029 | 0.0068 | 0.0019 | 0.0074 | 0.0098 | 0 |

**Table S4.** Outlier SNPs at genomic regions strongly associated with ecological adaptation in NW Atlantic herring (separate Microsoft Excel file).

**Table S5.** RDA model and ANOVA tests of the significance of the RDA model, axes, and environmental variables included in the model.

**RDA model**

Call: rda(formula = poolData_infoReg_final ~ dayLightHours + SST_Summer + SST_Winter + SST_spawn, data = envData_final_sc_set, scale = FALSE)

Inertia Proportion Rank

Total 822.7839 1.0000

Constrained 554.9930 0.6745 4

Unconstrained 267.7908 0.3255 8

Inertia is variance

Eigenvalues for constrained axes:

RDA1 RDA2 RDA3 RDA4

483.2 27.9 25.3 18.6

Eigenvalues for unconstrained axes:

PC1 PC2 PC3 PC4 PC5 PC6 PC7 PC8

100.21 35.92 28.35 25.09 21.08 20.05 19.25 17.83

**Test of significance of the model**

Permutation test for rda under reduced model

Permutation: free

Number of permutations: 999

Model: rda(formula = poolData_infoReg_final ~ dayLightHours + SST_Summer + SST_Winter + SST_spawn, data = envData_final_sc_set, scale = FALSE)

Df Variance F Pr(>F)

Model 4 554.99 4.145 0.003 **

Residual 8 267.79

---

Signif. codes: 0 ‘***’ 0.001 ‘**’ 0.01 ‘*’ 0.05 ‘.’ 0.1 ‘ ’ 1

**Test of significance of the RDA axes**

Permutation test for rda under reduced model

Forward tests for axes

Permutation: free

Number of permutations: 999

Model: rda(formula = poolData_infoReg_final ~ dayLightHours + SST_Summer + SST_Winter + SST_spawn, data = envData_final_sc_set, scale = FALSE)

Df Variance F Pr(>F)

RDA1 1 483.20 14.4350 0.003 **

RDA2 1 27.88 0.8328 0.974

RDA3 1 25.27 0.7549 0.950

RDA4 1 18.65 0.5571 0.910

Residual 8 267.79

---

Signif. codes: 0 ‘***’ 0.001 ‘**’ 0.01 ‘*’ 0.05 ‘.’ 0.1 ‘ ’ 1

**Test of significance of the environmental variables**

Permutation test for rda under reduced model

Marginal effects of terms

Permutation: free

Number of permutations: 1000

Model: rda(formula = poolData_infoReg_final ~ dayLightHours + SST_Summer + SST_Winter + SST_spawn, data = envData_final_sc_set, scale = FALSE)

Df Variance F Pr(>F)

dayLightHours 1 20.739 0.6195 0.521479

SST_Summer 1 26.536 0.7927 0.428571

SST_Winter 1 251.736 7.5204 0.004995 **

SST_spawn 1 25.522 0.7625 0.469530

Residual 8 267.791

---

Signif. codes: 0 ‘***’ 0.001 ‘**’ 0.01 ‘*’ 0.05 ‘.’ 0.1 ‘ ’ 1

**Table S6.** Ten genomic regions showing highly significant genetic differentiation among the northwest Atlantic herring populations. Description of the number and percentage of novel and shared SNPs between West and East Atlantic oceanic populations.

| **Genomic region** | **Characteristics** | **Population contrast** | **Novel locus** | **Total number of SNPs** | **Total number of outlier SNPs*** | **Number of novel outlier SNPs*** | **Number of shared^+^ outlier SNPs*** | **% of novel SNPs** | **% of shared SNPs** |
| --- | --- | --- | --- | --- | --- | --- | --- | --- | --- |
| chr8:23,040,136-30,729,461 | Undescribed structural variant | Spring vs Fall | No | 102739 | 1356 | 1349 | 7 | 0.995 | 0.005 |
| chr12:15,081,429-16,739,133 | *myhc* | Spring vs Fall | No | 4764 | 270 | 269 | 1 | 0.996 | 0.004 |
| chr15:6,750,000-7,000,000 | *rab15-sipa1l1* | Spring vs Fall | Yes | 854 | 3 | 3 | 0 | 1.000 | 0.000 |
| chr15:7,650,000-7,850,000 | *sox11b* | Spring vs Fall | No | 666 | 5 | 3 | 2 | 0.600 | 0.400 |
| chr15:8,800,000-9,075,000 | *tshr* | Spring vs Fall | No | 868 | 180 | 16 | 164 | 0.089 | 0.911 |
| chr15:9,200,000-9,350,000 | *flrt2-lncRNA* | Spring vs Fall | No | 715 | 15 | 13 | 2 | 0.867 | 0.133 |
| chr15:10,850,000-11,100,000 | *esr2a-syne2-lncRNA-l3hypdh* | Spring vs Fall | No | 943 | 132 | 73 | 59 | 0.553 | 0.447 |
| chr19:20,235,179-20,630,966 | *herpud2* | Spring vs Fall | No | 1534 | 167 | 16 | 151 | 0.096 | 0.904 |
| chr19:23,170,000-23,954,060 | *lncRNA-sgk-like* | Spring vs Fall | Yes | 5968 | 4 | 4 | 0 | 1.000 | 0.000 |
| chr12:17,823,410-25,605,433 | chr12 inversion | Northern vs Southern Fall | No | 64240 | 434 | 51 | 383 | 0.118 | 0.882 |

(*) For the Spring vs Fall contrast, outlier SNPs correspond to those with dAF >= 0.55, and for the Northern vs Southern Fall contrast, outlier SNPs are those with dAF >= 0.45.

(+) Shared SNPs were defined as outlier SNPs with a known association with adaptation as previously reported in Han et al. (2020).

**Figure S1**. Proportion of individuals at various gonadal maturity stages at the time of collection in each of the sampling sites. Gonadal maturity categories: 3 = Mid maturation, 4 = Late maturation, 5 = Spawning capable, 6 = Spawning, 7 = Spent-recovery, 8 = Abnormal (Bucholtz, Tomkiewicz, & Dalskov, 2008).


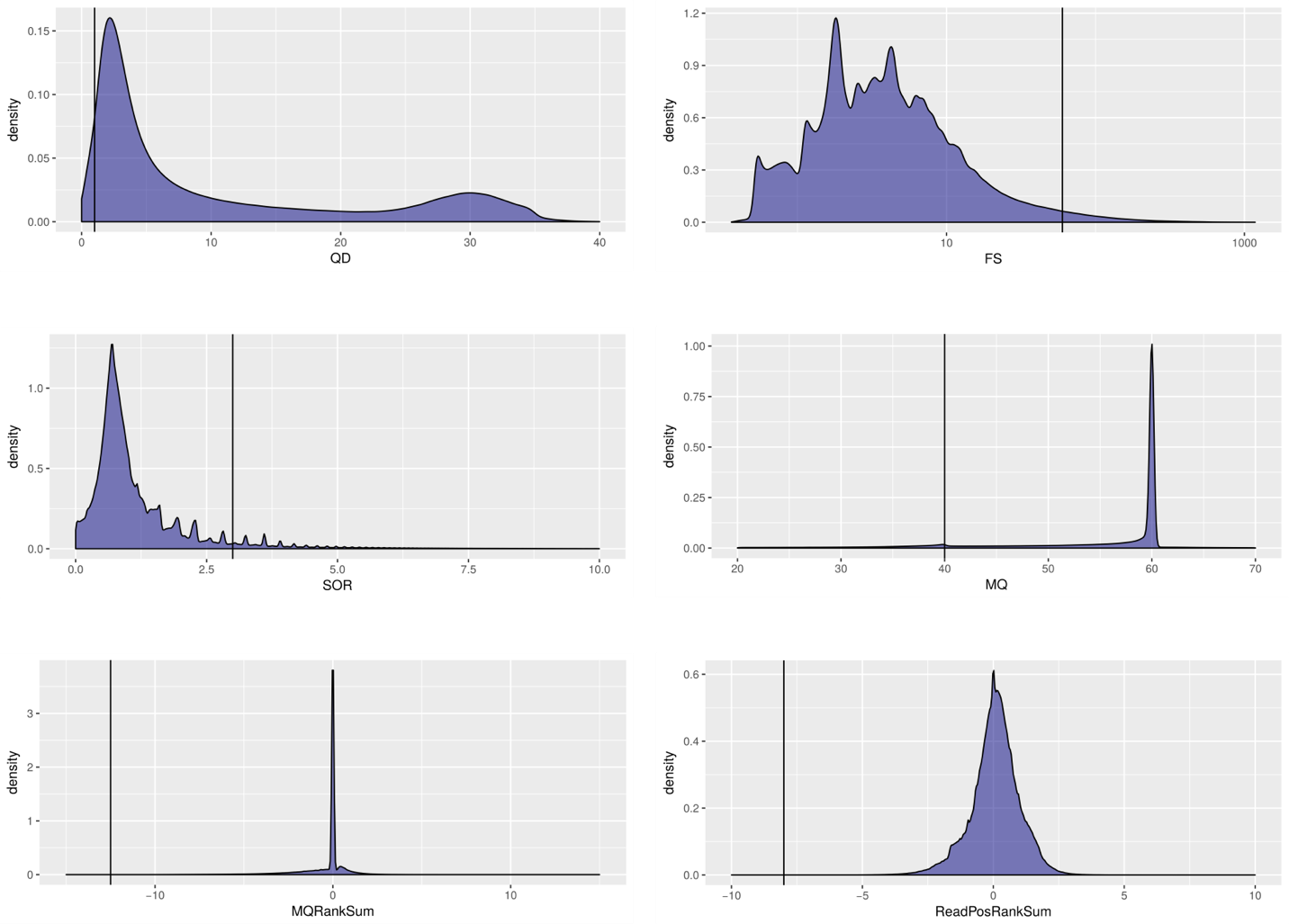


**Figure S2.** Density plots of GATK variant annotations used as reference to determine cutoff values to apply hard filters to raw SNP calls. The black vertical line shows the cutoff value used. Abbreviations: QualByDepth (QD) 2.0, FisherStrand (FS) 60.0, StrandOddsRatio (SOR) 3.0, RMSMappingQuality (MQ) 40.0, MappingQualityRankSumTest (MQRankSum) -12.5, ReadPosRankSumTest (ReadPosRankSum) -8.0. A complete explanation of each of these filters can be found in (Broad Institute, 2016).

**Figure S3**. Depth of coverage distribution per pool. The vertical lines indicate various measures of central tendency and cut off values. Dashed gray line: mean value, dashed purple line: mean ± 1 standard deviation (SD); gray continuous line: mode, red continuous line: mode ± 1/2 mode; dashed light blue line: minimum coverage 20X and maximum coverage 3 times the mean value; dashed gold line: quantile 5-99%.

**Figure S4**. Empirical distribution of pool-allele frequencies and they standard deviation. The dashed red lines indicate cut of values to determine the set of undifferentiated markers (with 0.03 < allele frequency SD ≤ 0.09) and highly differentiated markers (allele frequency SD ≥ 0.2 from the mean).

**Figure S5.** Pairwise correlation of the initial set of environmental variables considered for environment association analyses. Below the diagonal a scatterplot for each pairwise comparison is shown, and above the diagonal the correspondent Pearson correlation coefficients are presented; their font size reflects their magnitude.

**Figure S6.** Pairwise correlation of the final set of environmental variables considered for environment association analyses. Below the diagonal a scatterplot for each pairwise comparison is shown, and above the diagonal the correspondent Pearson correlation coefficients are presented; their font size reflects their magnitude. The uncorrelated environmental variables were: sea surface summer temperature (SST_Summer_), sea surface winter temperature (SST_Winter_), sea surface temperature at spawning (SST_spawn_), and day light hours (dayLightHours).


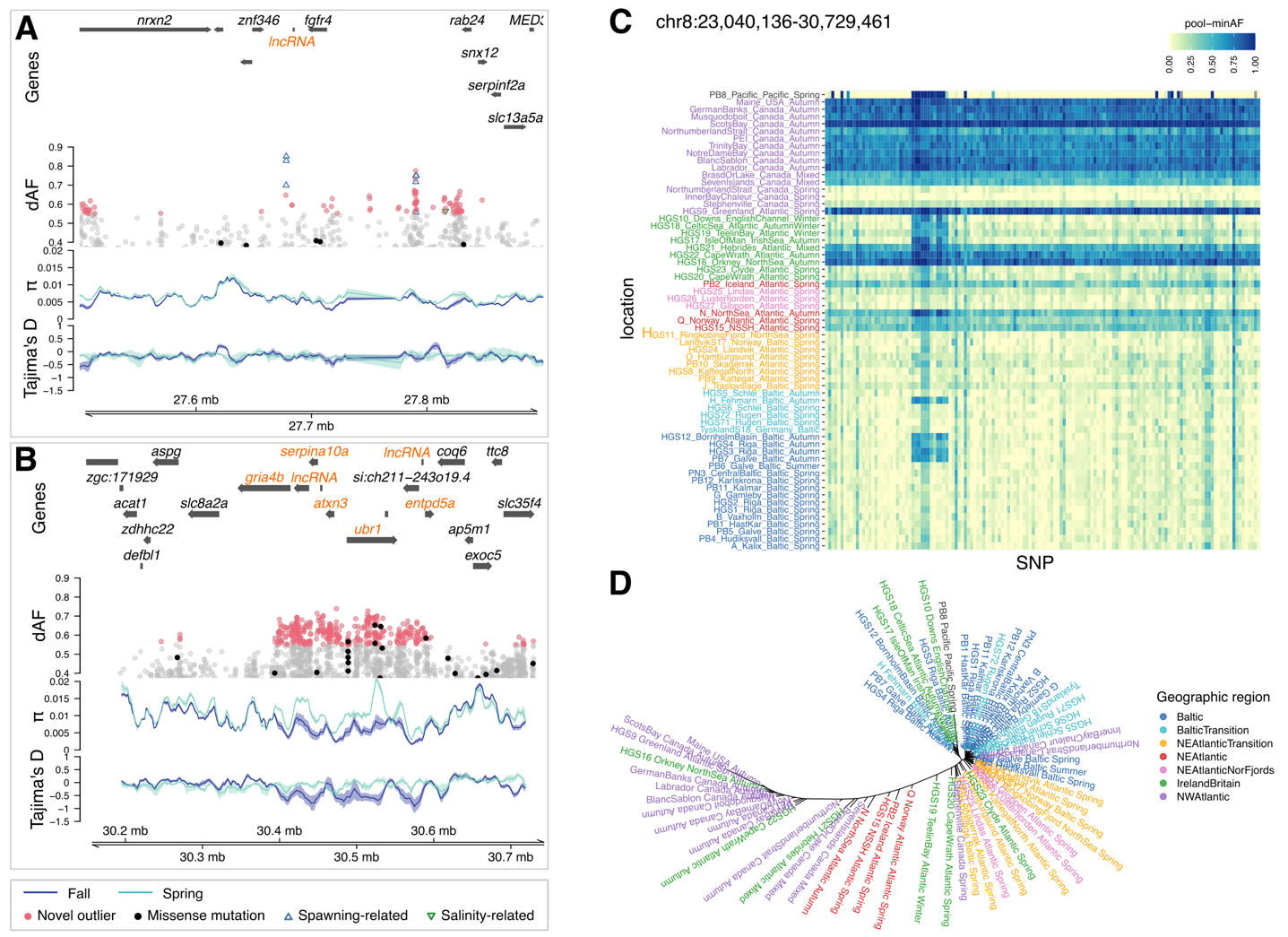


**Figure S7**. Selection signals on the putative inversion on chromosome 8. Zoom-in to two regions, (**A**) chr8:27,500,000-27,900,000, which contains the most differentiated SNPs, and (**B**) chr8:30,150,000-30,750,000, which shows a putative selective sweep among fall spawners. Each zoom-in plot consists of four tracks, from top to bottom: gene modes, genes of interest are highlighted in orange. Genetic differentiation between spring and fall spawners for SNPs with dAF ≥ 0.4. Novel outlier SNPs (dAF ≥ 0.55) are denoted as red filled circles, missense mutations as filled black circles, spawning-related SNPs as empty blue triangles, and other SNPs are gray circles. Average nucleotide diversity (π) and Tajima’s D (window size 10 Kbp, step size 2 Kbp) for spring and fall spawners, in light and dark blue lines, respectively. (**C**) Heatmap plot depicting the minor pool-allele frequency per location (rows) for diagnostic SNPs (columns). (**D**) Neighbor-joining tree based on diagnostic genetic variants (dAF >= 0.55).


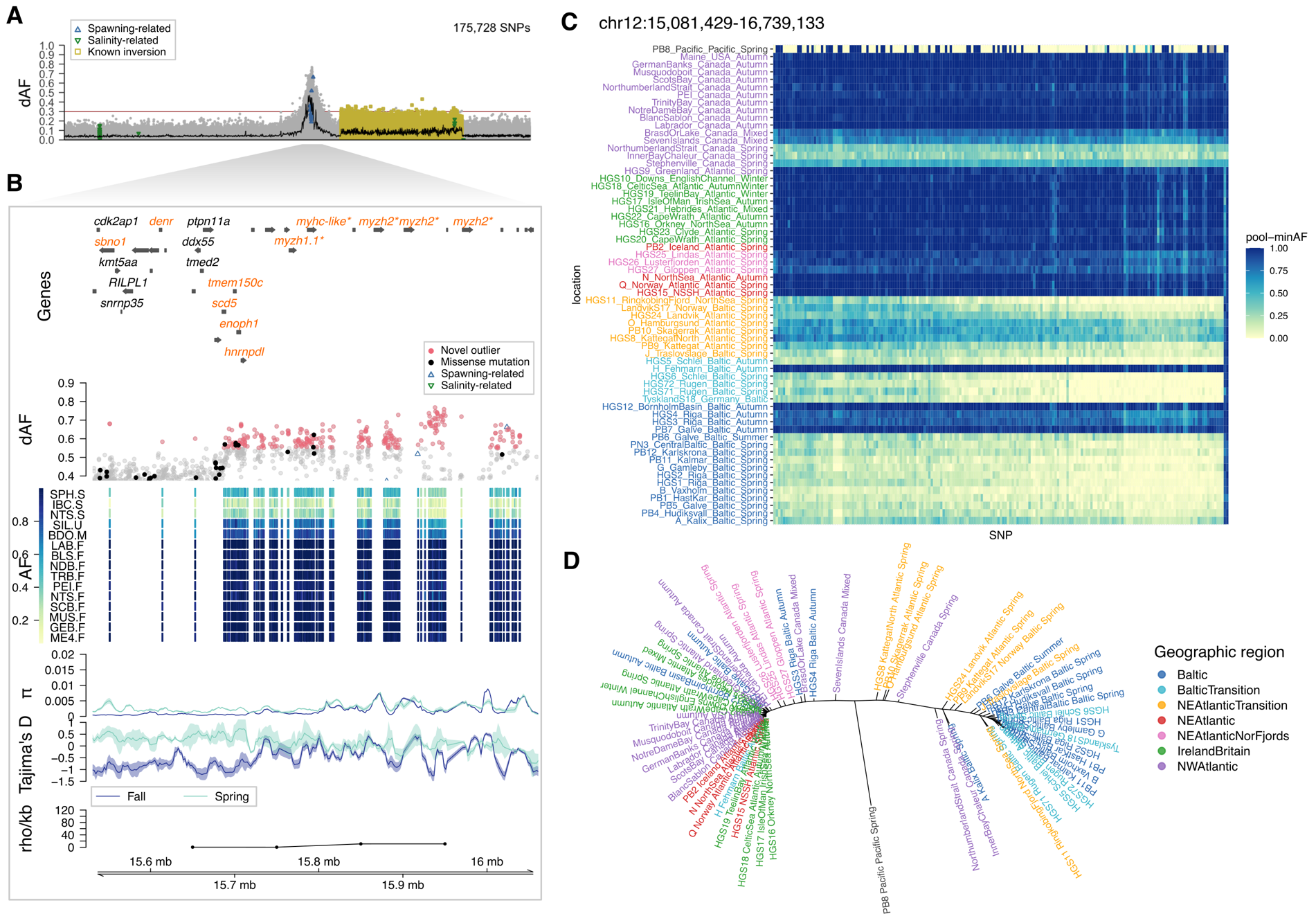


**Figure S8**. Selection signal on chromosome 12. (**A**) Genetic differentiation (dAF) along chr 12. (**B**) Close-up to the target region. This plot consists of five tracks, from top to bottom: gene models; genetic differentiation between spring and fall spawners for SNPs with dAF ≥ 0.4. Novel outlier SNPs (dAF ≥ 0.55) are denoted as red filled circles, missense mutations as filled black circles, spawning-related SNPs as empty blue triangles, and other SNPs are gray circles; heatmap plot depicting the minor allele frequency per population (rows) for the novel outlier SNPs (columns); average nucleotide diversity (π) and Tajima’s D (window size 10 Kbp, step size 2 Kbp) for spring and fall spawners, in light and dark blue lines, respectively; and estimate of recombination rate (rho/Kbp) every 100 Kbp (Pettersson et al., 2019). (**C**) Heatmap plot depicting the minor pool-allele frequency per location (rows) for diagnostic SNPs (columns). (**D**) Neighbor-joining tree based on diagnostic genetic variants (dAF >= 0.55).


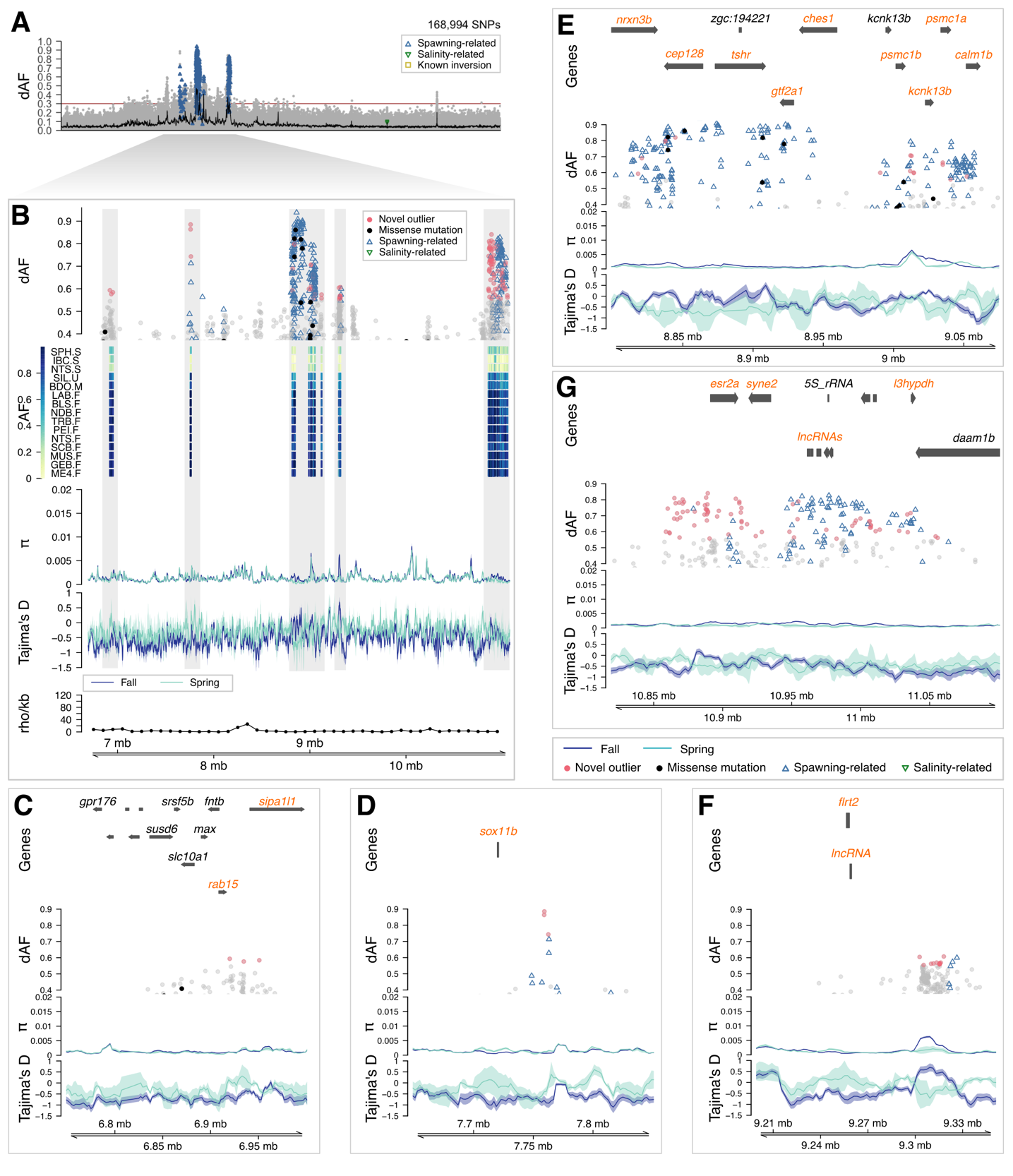


**Figure S9**. Close up to the selection signal on chr 15. (**A**) Genetic differentiation (dAF) along the chromosome. (**B**) Close-up to the target region. This plot consists of five tracks, from top to bottom: genetic differentiation between spring and fall spawners for SNPs with dAF ≥ 0.4. Novel outlier SNPs (dAF ≥ 0.55) are denoted as red filled circles, missense mutations as filled black circles, spawning-related SNPs as empty blue triangles, and other SNPs are gray circles; heatmap plot depicting the minor allele frequency per population (rows) for the novel outlier SNPs (columns); average nucleotide diversity (π) and Tajima’s D (window size 10 Kbp, step size 2 Kbp) for spring and fall spawners, in light and dark blue lines, respectively; and estimate of recombination rate (rho/Kbp) every 100 Kbp (Pettersson et al., 2019). Zoom-in plots to 5 loci within this region, (**C**) chr15:6,750,000-7,000,000, (**D**) chr15:7,650,000-7,850,000, (**E**) chr15:8,540,000-9,070,000, (**F**) chr15:9,200,000-9,350,000, (**G**) chr15:10,820,000-11,100,000. Each plot has four tracks: gene models, dAF, nucleotide diversity, and Tajima’s D.


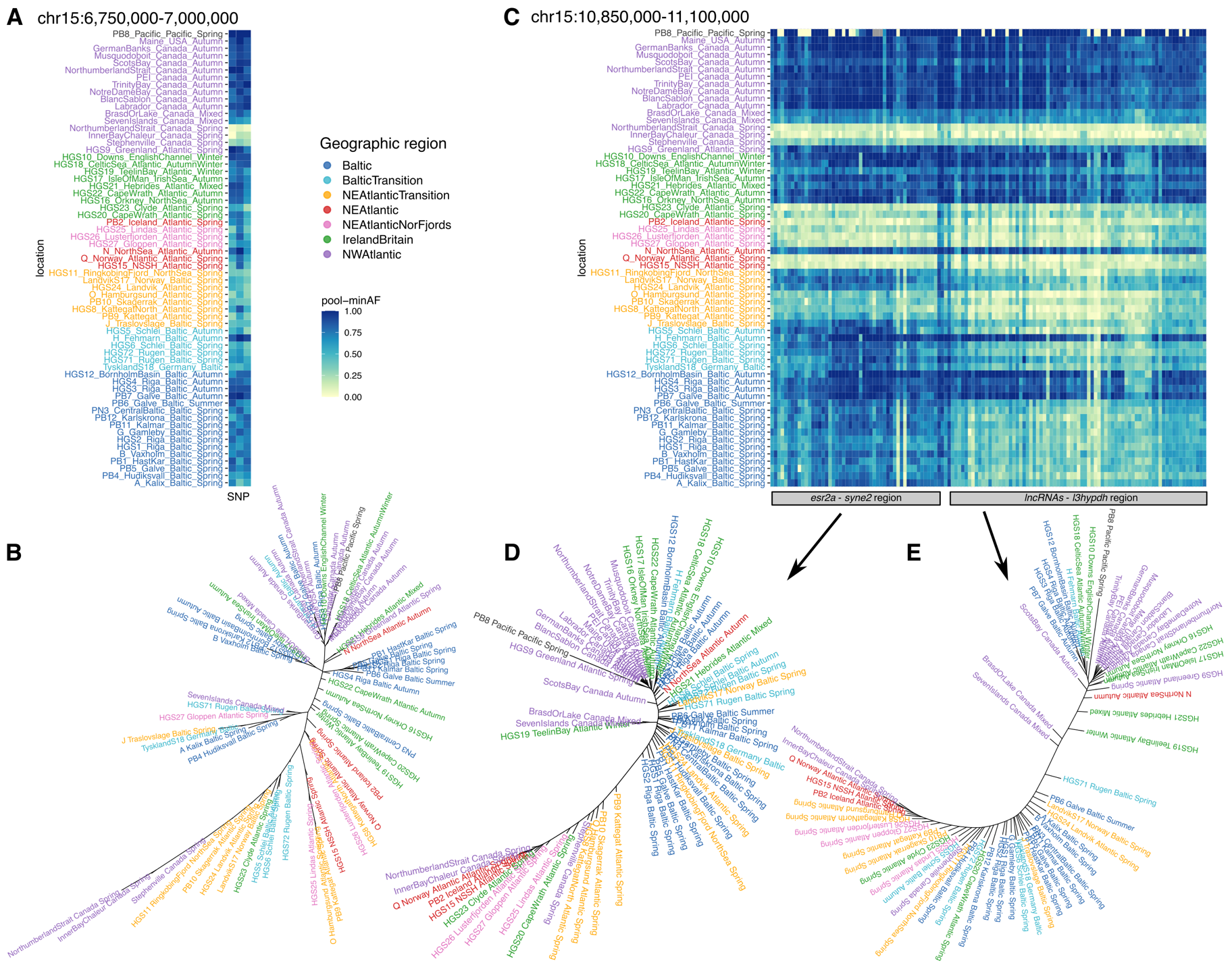


**Figure S10**. Comparison of the genetic patterns in two outlier regions in chr 15. The first region (**A-B**) harbors the *rab15-si* gene. (**A**) Pool-allele frequencies and (**B**) neighbor-joining tree based on the novel genetic variants (dAF > 0.55). The second region (**C-E**) comprises the *esr2a*-*syne2-lncRN-l3hypdh* genes. (**C**) Pool-allele frequencies of outlier SNPs for the entire region. neighbor-joining tree (**D**) for the novel genetic variants in the *esr2a*-*syne2* region, and in the (**E**) *lncRN-l3hypdh* region. Note that the sample from Greenland corresponds to summer spawners as indicated in the Supplementary file 1 of Han et al. (2020).


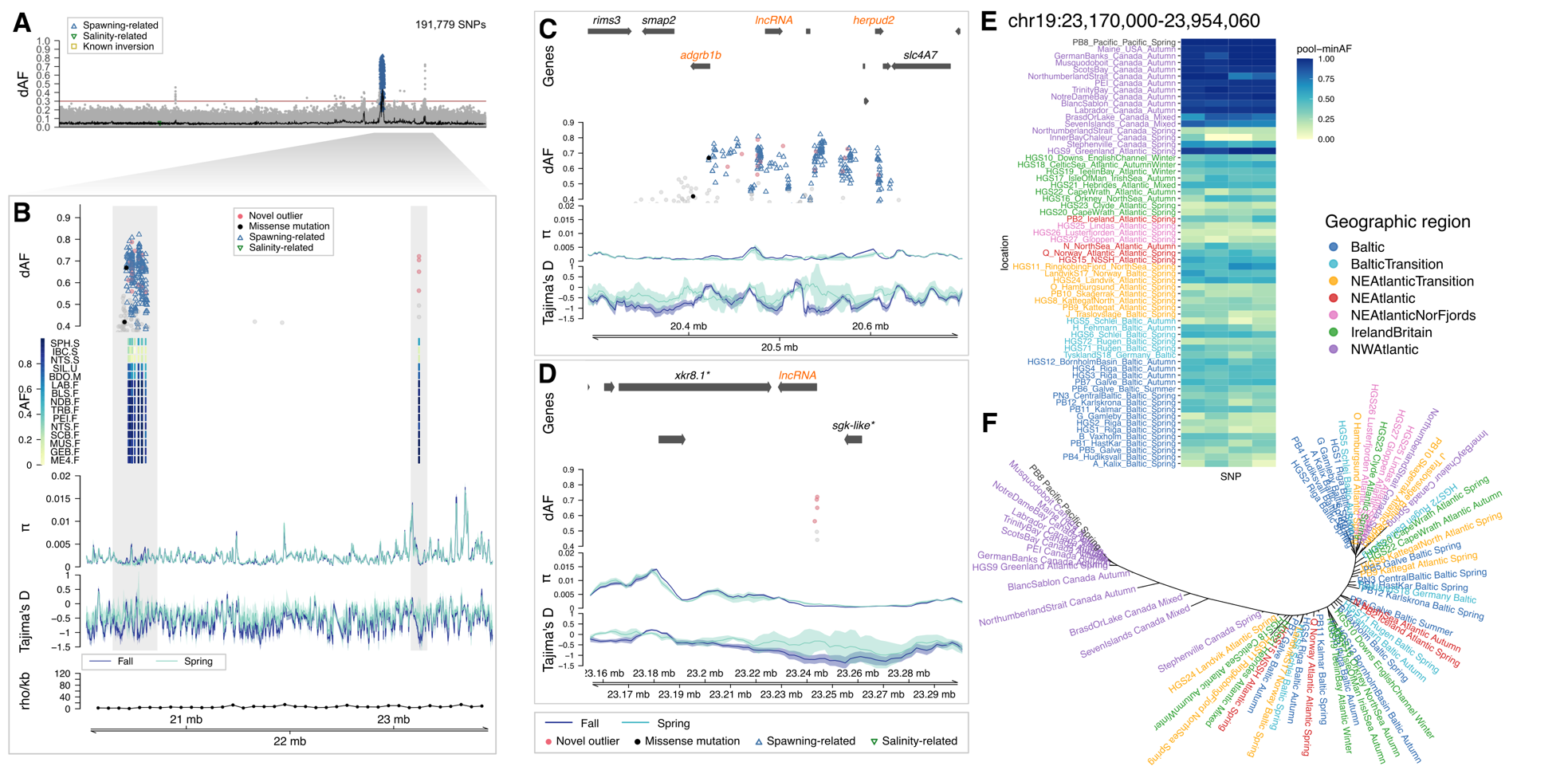


**Figure S11**. Selection signal on chr 19. (**A**) Genetic differentiation (dAF) along the chromosome. (**B**) Close-up to the target region. This plot consists of five tracks, from top to bottom: genetic differentiation between spring and fall spawners for SNPs with dAF ≥ 0.4. Novel outlier SNPs (dAF ≥ 0.55) are denoted as red filled circles, missense mutations as filled black circles, spawning-related SNPs as empty blue triangles, and other SNPs are gray circles; heatmap plot depicting the minor allele frequency per population (rows) for the novel outlier SNPs (columns); average nucleotide diversity (π) and Tajima’s D (window size 10 Kbp, step size 2 Kbp) for spring and fall spawners, in light and dark blue lines, respectively; and estimate of recombination rate (rho/Kbp) every 100 Kbp (Pettersson et al., 2019). Zoom-in plots to 5 loci within this region, (**C**) 19:20,290,000-20,700,000, and (**D**) 19:23,155,000-23,300,000. Each plot has four tracks: gene models, dAF, nucleotide diversity, and Tajima’s D. (**E**) Pool-allele frequencies of diagnostic SNPs (dAF >= 0.55). (**F**) neighbor-joining tree based on these genetic variants. Note that the sample from Greenland corresponds to summer spawners as indicated in the Supplementary file 1 of Han et al. (2020).

**
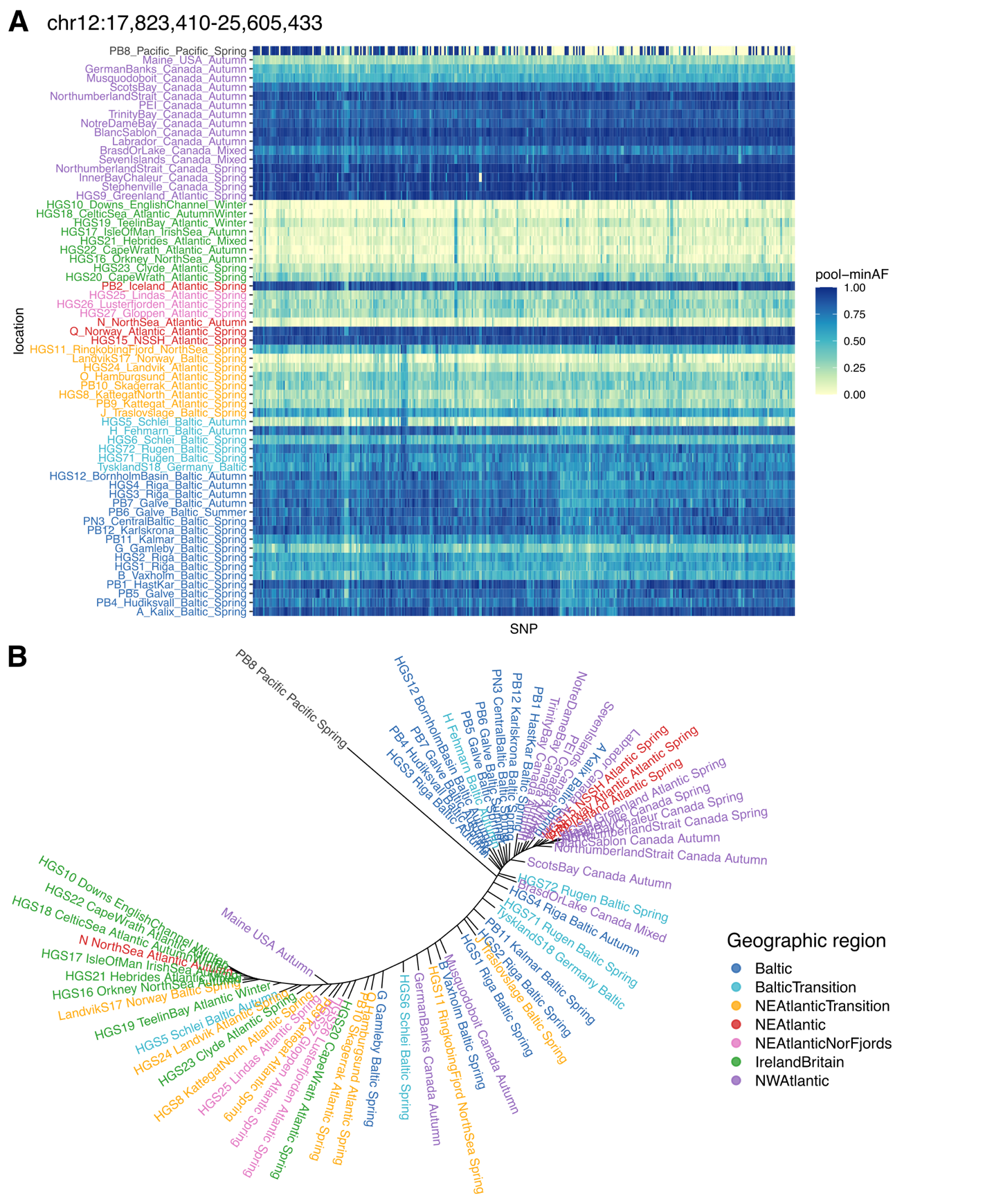
**

**Figure S12**. Selection signal on chr 12 corresponding to a known chromosomal inversion. (**A**) Heatmap plot depicting the minor pool-allele frequency per location (rows) for diagnostic SNPs (columns). (**B**) Neighbor-joining tree based on diagnostic genetic variants (dAF >= 0.45). Note that the sample from Greenland corresponds to summer spawners as indicated in the Supplementary file 1 of Han et al. (2020).

**
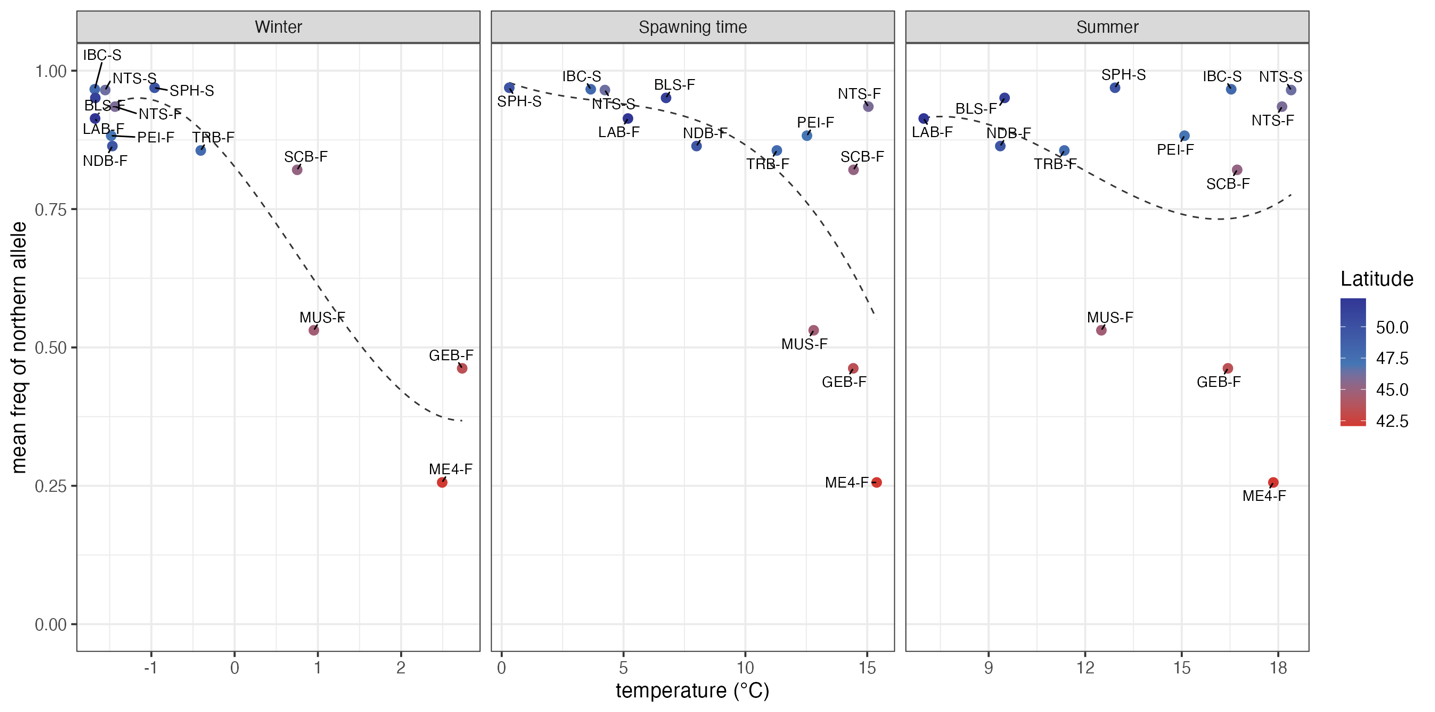
**

**Figure S13.** Variation of the mean frequency of the northern allele of the chr12 inversion with average seawater temperature (°C) per location over the winter, spawning time, and summer months. Each dot represents a location, and its color indicate its latitude (N°). The dashed lined shows the fitted polynomial liner regression.

**Figure S14.** Average sea water salinity (PSU) per location. Blue bars indicate values for the summer months (SST_Summer_), orange bars show values for the winter months (SST_Winter_), and blue bars are for the spawning month (SST_spawn_).

**REFERENCES**

Broad Institute. (2016). Understanding and adapting the generic hard-filtering recommendations. Retrieved May 20, 2018, from https://gatkforums.broadinstitute.org/gatk/discussion/6925/understanding-and-adapting-the-generic-hard-filtering-recommendations

Bucholtz, R. H., Tomkiewicz, J., & Dalskov, J. (2008). Manual to determine gonadal maturity of herring (Clupea harengus L.). In *DTU Aqua-report*. Retrieved from http://scholar.google.com/scholar?hl=en&btnG=Search&q=intitle:Manual+to+determine+gonadal+maturity+of+herring+(Clupea+harengus+L.)#0
